# A Cdk5 inhibitor restores cognitive function and alleviates type 2 diabetes in mice

**DOI:** 10.1016/j.isci.2025.112200

**Published:** 2025-03-11

**Authors:** Sangita Paul, Remya Chandran, Dileep K. Vijayan, Juhi Bhardwaj, Praveen Singh, Poornima Shetty, Srinivas Cheruku, Sajith Meleveetil, Binukumar Balachandran Krishnamma

**Affiliations:** 1 CSIR Institute of Genomics and Integrative Biology, New Delhi, India; 2 Academy of Scientific and Innovative Research (AcSIR), Ghaziabad 201002, India; 3 Laboratory for Computational and Structural Biology, Jubilee Centre for Medical Research, Jubilee Mission Medical College and Research Institute, Thrissur 680005, Kerala, India; 4 Srinivasa Engineering College, Mukka, Mangalore 574146, India; 5 Department of Chemistry, Manasa Gangotri, Mysore University, Mysuru 570005, India; 6 Department of Chemistry, SSIT, Sri Siddhartha Academy of Higher Education, Tumkur 572107, Karnataka, India

**Keywords:** Pharmacology, Natural sciences, Biological sciences, Physiology, Neuroscience, Endocrinology

## Abstract

Type 2 diabetes (T2D) is a metabolic disorder commonly linked with cognitive decline, increasing patients’ susceptibility to dementia. Alzheimer’s disease (AD) has a strong connection with hyperglycemia and insulin dysregulation. Interestingly, certain anti-diabetic drugs have shown potential in reducing T2D-induced cognitive impairment. Previous studies, including ours, have highlighted the dysregulation of cyclin-dependent kinase 5 (Cdk5) activity in both T2D and AD, which may contribute to pathological changes in these conditions. Thus, targeting the Cdk5 kinase could offer a therapeutic approach for T2D and cognitive deterioration. Our research identifies Cdk5 as a key link between T2D and cognitive decline. By screening the KINACore library, we discovered two new brain-penetrant Cdk5 inhibitors, BLINK11 and BLINK15. In a high-fat diet-induced T2D model, these inhibitors improved blood glucose levels, obesity, and cognitive function. BLINK11, in particular, shows promise as a therapeutic candidate for treating cognitive impairment associated with T2D.

## Highlights

• Cdk5 dysregulation is a critical factor in cognitive decline associated with T2D and AD
• BLINK11 and BLINK15 cross BBB, offering anti-diabetic and neuroprotective benefits
• BLINK11 lowers blood glucose, enhances cognition, and reduces neurodegeneration in T2D mice
• Cdk5 inhibition via BLINK11 may help treat T2D and related cognitive impairment

## Graphical abstract

**Figure:**
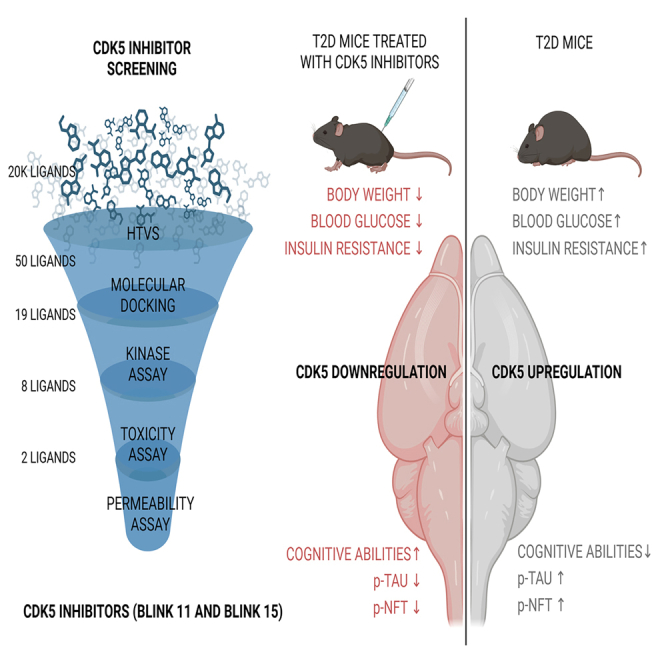

## Introduction

Diabetes is a long-term metabolic illness, and patients with type 2 diabetes (T2D) have been shown to experience a wide range of neurological abnormalities, including memory loss and cognitive decline.^1^^,^^2^ Epidemiologically it has been proven that type 2 diabetic patients have a 70% increased risk of developing Alzheimer’s-like phenotypes such as neuroinflammation and cognitive impairment.^3^^,^^4^^,^^5^ Insulin dysregulation has negative impacts on both peripheral glucose regulation and brain function since insulin is essential for both sustaining normal brain function and peripheral glucose metabolism. Numerous studies have indicated that an estimated two-to-three-fold relative risk for AD is associated with insulin resistance, which is characterized by unsuccessful glucose utilization.^6^ This relationship may be explained by several variables, including oxidative stress, inflammation, mitochondrial dysfunction, and insulin-degrading enzyme (IDE) activity. Cyclin-dependent kinase 5 (Cdk5) can be a potent link between T2D and cognitive impairment as this kinase plays a critical role in both disease types.^7^^,^^8^ Studies have shown inhibition of hyperactive Cdk5 mitigates intracerebral neurotoxicity in diabetes and attenuates neuronal death and cognitive impairments.^9^^,^^10^

Cdk5 is an atypical cyclin-dependent kinase that gets activated mainly through p35 and p39, predominantly in neuronal cells.^11^ Its related downstream signaling pathways are known to strongly participate in several neurological events regulating neurodevelopment as well as neurodegeneration and cognitive impairment in the brain.^12^^,^^13^^,^^14^ Cdk5 contributes to neurodevelopment and neuronal migration by phosphorylating various microtubules, actin-associated proteins, and cell motility proteins.^15^^,^^16^ Again, cellular stress causes calcium influx into the cells enhancing calpain to cleave p35 into a truncated activator protein p25 that can more stably bind to Cdk5 and causes its hyperactivation. Contrastingly, p25 alters the substrate specificity of Cdk5 and^16^ favors its contribution to neurotoxicity. Partial knockdown of Cdk5 or inhibition of Cdk5 activity is considered a therapeutic strategy to rescue neurological disorders.^15^^,^^16^^,^^17^

Interestingly studies have also shown the presence of Cdk5 and its activator p35 in pancreatic beta cells. The presence of glucose increases the expression of the p35 gene, which in turn encourages the creation of active Cdk5/p35 complexes controlling the expression of the insulin gene.^18^^,^^19^ A prolonged increase in glucose causes hyperactivation of Cdk5 that results in the inadequate release of insulin by pancreatic beta cells.^19^ Therefore, dysregulation of Cdk5 is reported in T2D conditions and Cdk5 inhibition also shows antidiabetic effects.^9^ However, screening a Cdk5 inhibitor that efficiently inhibits Cdk5 can help rescue cognitive impairment induced by T2D in diabetic models.

## Results

### HTVS combined with MD simulations identified putative Cdk5 inhibitors

**Figure 1:**
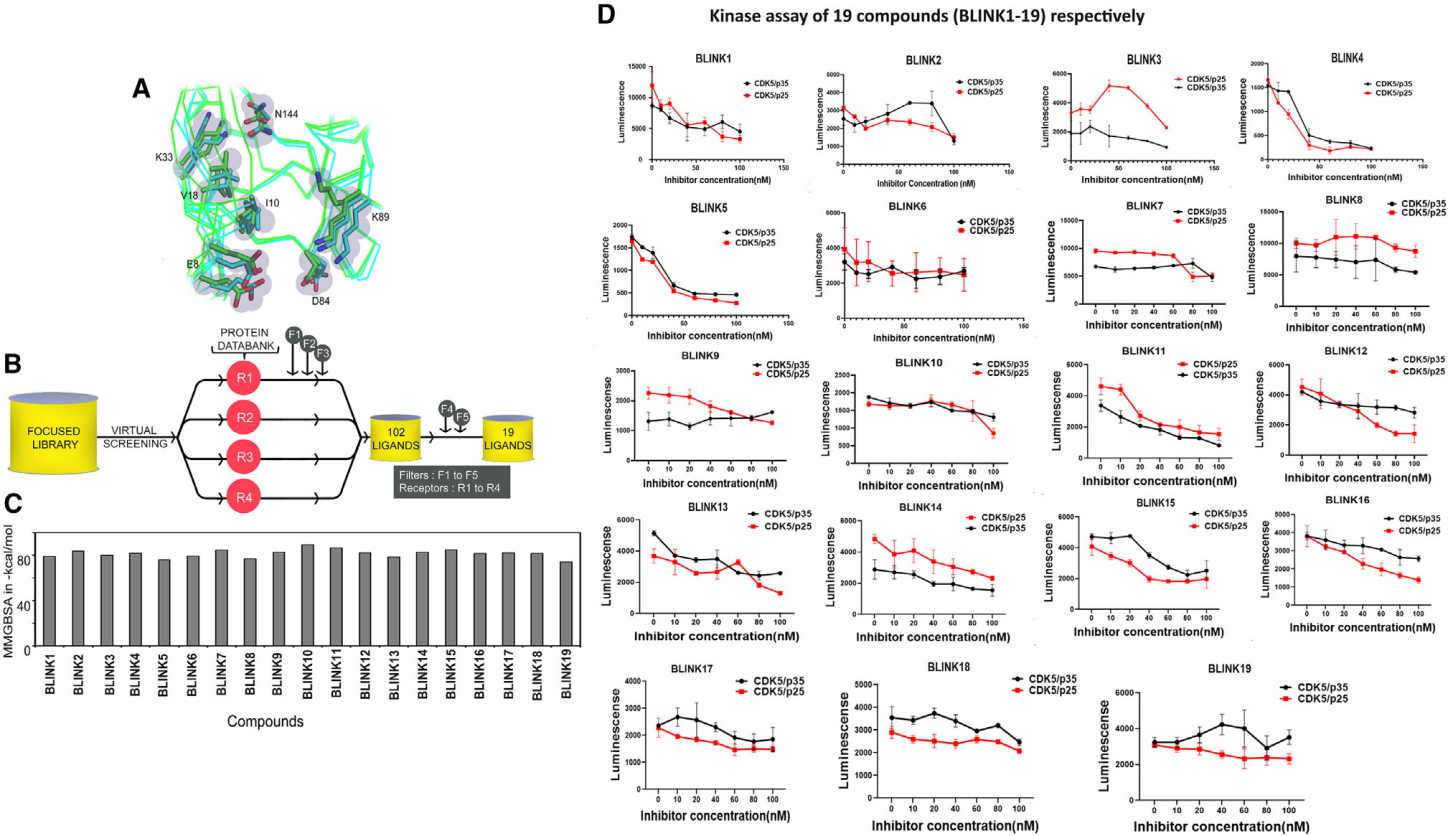
Virtual screening of Cdk5 inhibitors (A) Superposition of different Cdk5 structures. The residues displaying distinct conformations are depicted in a stick model, while the protein backbone is illustrated using a ribbon model. Overview of the virtual screening workflow. (B) The ligands from the KINACore Library were screened against four distinct protein structures that differ in the orientation of the sidechains: PDB IDs 1UNH, 1UNL, 3O0G, and 4AU8, denoted as R1–R4. These four receptors were employed for high-throughput virtual screening. The screening process comprised multiple filters, namely F1–F5. F1, F2, and F3 corresponded to diverse docking protocols, specifically HTVS, SP, and XP docking, respectively. Additionally, F4 and F5 incorporated MM-GBSA calculations and visual inspections, respectively. (C) The binding energies of the final 19 ligands (BLINK1 to BLINK19) were determined using the MM-GBSA method. (D) Cdk5 activity *in vitro* with inhibitors in increasing concentration. Graphs 1–19 represent Cdk5 assay with p35 and 25 as activators with six different concentrations of BLINK1-19 (10–100 nM). IC50 was calculated for individual inhibitors with Cdk5/p35 and Cdk5/p25 complexes respectively. All the assays were performed in triplicates and 3 sets of data were considered for the analysis purpose.

The compounds in the KINACore Library were designed based on two criteria: (1) molecules that share high structural similarity with known kinase activators and (2) molecules that have high structural similarities with the adenosine portion of ATP. The critical analysis of all Cdk5 structures revealed multiple conformations in the proximity of the active site, especially on the residues like E8, I10, K89, and N144 (Figure 1A). As all the structures are ligand-bound, we assumed that these conformational variations might exert an influence on ligand binding. Similarly, the receptor structures that are selected for our modeling studies include the following: (1) Cdk5-p25 in complex with indirubin (PDB ID: 1UNH), referred as R1; Cdk5-p25 in complex with roscovitine (PDB ID: 1UNL), referred as R2; Cdk5-p25 in complex with an ATP analog (PDB ID: 3O0G), referred as R3; and Cdk5 in complex with compound 4a (PDB ID: 4AU8), referred as R4. The ligands name in Table S1 are indicated as L1-indirubin; L2-roscovitine; L3-{4-amino-2-[(4-chlorophenyl)amino]-1,3-thiazol-5-yl}(3-nitrophenyl)methanone; L4-4-(1,3-benzothiazol-2-yl)thiophene-2-sulfonamide (Table S1).

Our high-throughput virtual screening (HTVS) toward four receptor conformations identified 50 promising compounds for further studies (overall workflow of the *in-silico* studies shown in Figure 1B). Interestingly, we observed that the screening against R1 and R2 is more energetically favorable. It appears that the conformation of I10 in R3 and R4 is apparently affecting the binding of ligands, reflected in lower binding energy. Finally, out of the 50 ligands, we selected 19 ligands (Table S2) for the molecular dynamics (MD) simulations. All these 19 ligands were named after the lab as BLINK1–BLINK19, respectively; binding energies of these ligands are shown in Figure 1C. The structure of all the compounds is depicted in Figure S1.

**Figure 2:**
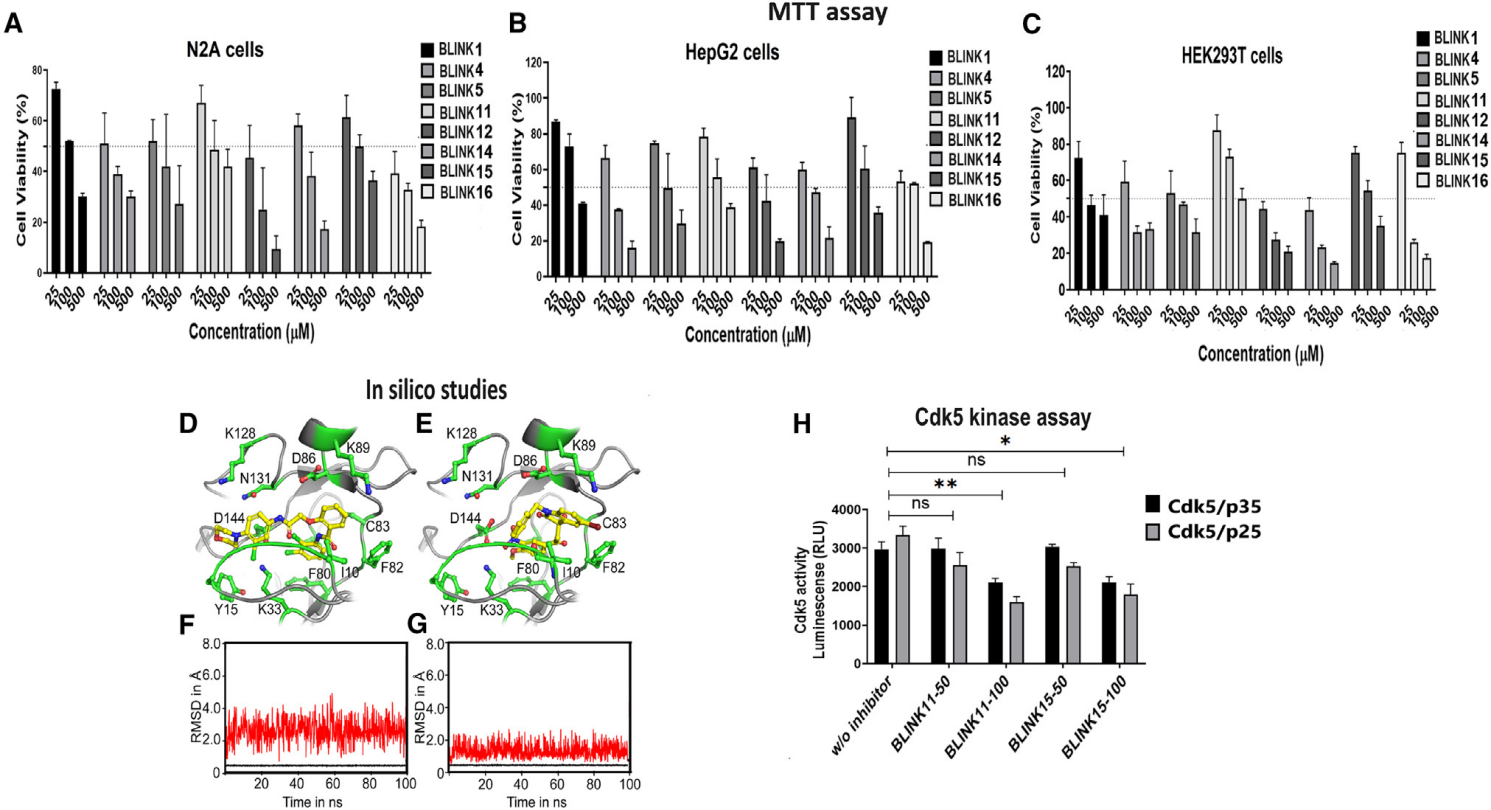
The cell viability test results for various inhibitors across different cell lines (A–C) The bar graph illustrates the percentage of cell viability, as determined by the MTT assay, for compounds BLINK1, BLINK4, BLINK5, BLINK11, BLINK12, BLINK14, BLINK15, and BLINK16 at concentrations of 25, 100, and 500 μM in (A) N2A cells, (B) HepG2 cells, and (C) HEK293T cells. All experiments were conducted in triplicates, and the data are presented as the mean ± standard deviation (SD). This analysis provides insights into the impact of these compounds on cell viability across different cell lines at varying concentrations. (D–G) Illustration of ligand binding modes within the ATP binding pocket of Cdk5: the orientations of (D) BLINK11 and (E) BLINK15 within the ATP binding pocket of Cdk5. The RMSD for BLINK11 (F) and BLINK15 (G) at the ATP binding pocket of Cdk5 over the course of a 100 ns simulation. The protein and the ligand RMSDs are shown in black and red lines, respectively. (H) The bar graph shows Cdk5 kinase activity in pulled down Cdk5 from cells overexpressed with Cdk5/p35 and Cdk5/p25 followed by BLINK11 and BLINK15 drug treatment at concentrations 50 nM and 100 nM. Two-way ANOVA with Turkey’s test were used for the analysis, and the values were provided as mean ± SD; *n* = 6, ∗*p* < 0.05, ∗∗*p* < 0.01; ∗∗∗*p* < 0.001 and ns denotes non-significant.

The MD simulation studies suggested that the ligands displayed versatile action on the active site of Cdk5 (Figures 2D and 2E; Figures S2A–S2Q).

### *In vitro* evaluation of putative Cdk5 inhibitors: Kinase assay, IC50 determination, and cell toxicity screening

**Scheme 1:**
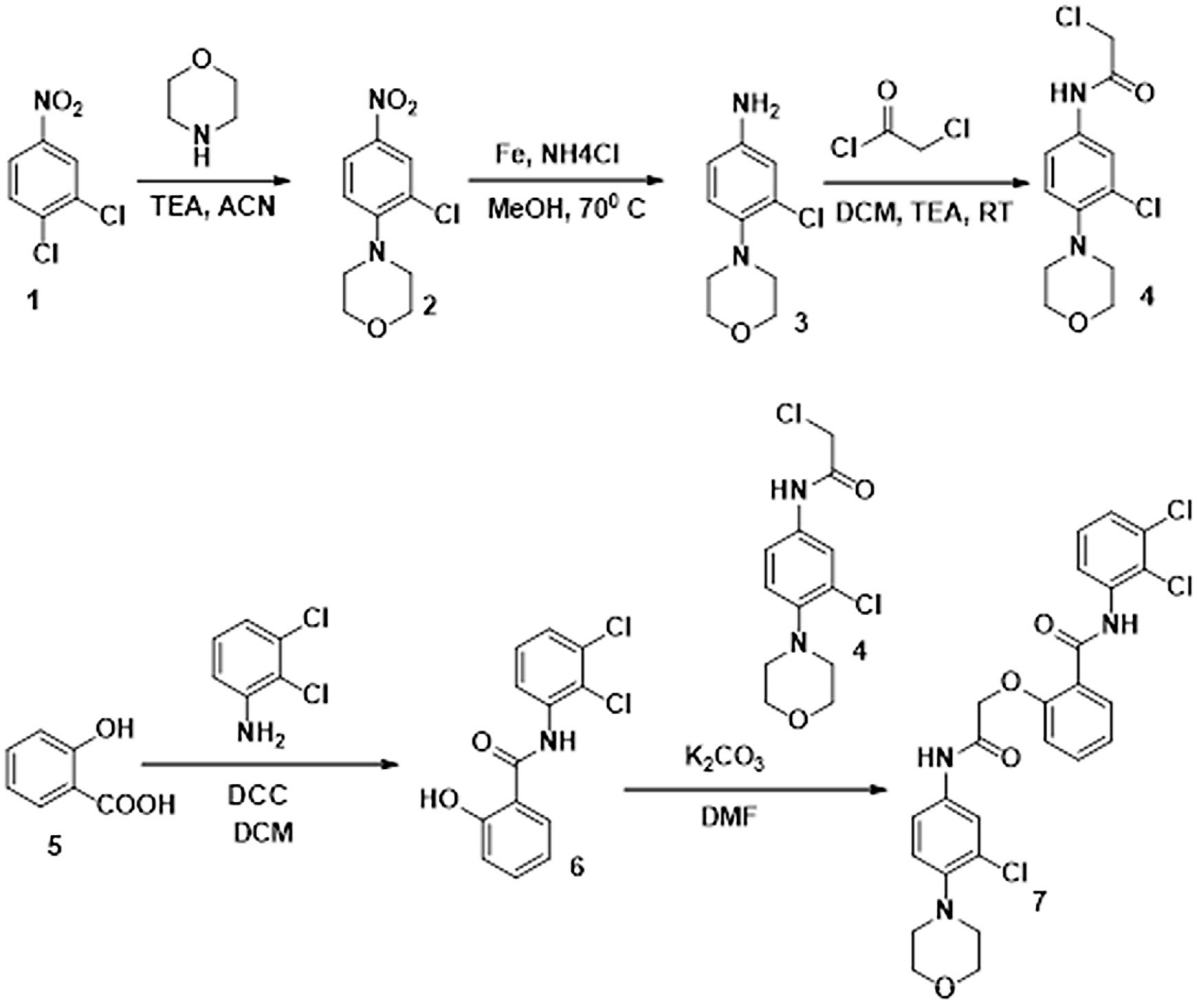
Synthesis of compound 7

The putative Cdk5 inhibitors were evaluated through an *in vitro* screening process using the Cdk5 kinase assay. The luminescence observed was directly proportional to Cdk5 activity. Figure 1D presents the kinase assay results for all 19 inhibitors at increasing concentrations (0–100 nM) for both Cdk5/p35 and Cdk5/p25 complexes. The calculated IC50 values are summarized in Table S3. Among the inhibitors tested, BLINK1, 4, 5, 11, 12, and 14–16 exhibited a more pronounced decrease in Cdk5 activity, suggesting their potential as effective inhibitors. These eight compounds were subjected to further screening based on cell toxicity using the MTT (3-(4,5-dimethylthiazol-2-yl)-2,5-diphenyltetrazolium bromide) assay. The MTT assay was conducted on three different cell lines: HEK293T, HepG2, and Neuro2A cells. The concentrations tested ranged from 25 to 100 μM (Figures 2A–2C) indicating that compounds BLINK11 (Scheme 1) and BLINK15 maintained cell viability above 50% at all doses. This observation suggests a favorable safety profile for these compounds in terms of cell toxicity. The study highlights the importance of not only inhibiting Cdk5 activity but also ensuring minimal cytotoxicity, and these two compounds demonstrated promising characteristics in both aspects. We, however, proceeded our study with the two compounds BLINK11 and BLINK15, as they exhibited a more pronounced decrease in Cdk5/p25 activity when compared to other BLINKS. BLNK11 and BLNK15 also exhibit relatively lower activity against the Cdk5/p35 complex (see Table S3).

### Enhanced *in vitro* activity of BLINK11 and BLINK15: Mechanistic insights from *in silico* studies

The enhanced activity of BLINK11 and BLINK15 was explained through computational approaches. Molecular docking analysis revealed specific interactions of these ligands within the ATP (Adinosine tri-phosphate) binding pocket of Cdk5 (Figures 2D and 2E). The CDK5 inhibitors typically form hydrogen bonds with E81 and C83, while they also engage in hydrophobic interactions with residues I10, Q85, and L133.^20^

Initially, BLINK11 forms a hydrogen bond with Q130 via its amide group but shifts to form a bond with C83 during MD simulations. Its morpholine ring also bonds with D144 of the DFG motif and interacts with D86 for over 85% of the simulation. An intramolecular hydrogen bond between its keto and amine groups adds conformational constraints. The dichloro phenyl benzamide moiety occupies the hinge region, facilitating interactions with C83, while the rest extends toward the DFG motif of the activation loop in Cdk5. BLINK11 also makes several hydrophobic interactions, involving residues like I10, C83, F82, L133, F80, V64, A31, A143, and V18. Figure 2D shows BLINK11 binding within the ATP binding pocket, while Figure 2F displays the root-mean-square deviation (RMSD) for BLINK11 and protein during the 100 ns simulation.^21^^,^^22^

The orientation of BLINK15 at the ATP binding pocket is shown in Figure 2G. BLINK15 has been found to exhibit a similar hydrogen-bonding pattern with C83 as observed in the case of other potent inhibitors of Cdk5. The carbonyl oxygen of C83 accepts an H-atom from the -OH group while the amide group donates an H-atom to the keto group of BLINK15. The interactions formed with C83 are found to be extremely stable during the MD simulation attributed to the inhibitory potency of BLINK15. In addition, the nitro group makes a salt bridge with K33 for ∼41% of the simulation time. The binding of BLINK15 was additionally stabilized by various hydrophobic interactions, similar to those observed in the case of BLINK11 contributing to the high binding stability of BLINK15 at the binding site of Cdk5 (Figure 2G).

### Cellular impact and BBB permeability assessment of Cdk5 inhibitors BLINK11 and BLINK15

Our *in vitro* and *in silico* studies clearly demonstrated the inhibitory effects of compounds BLINK11 and BLINK15 against CDK5. Expanding our research to a cellular context, we conducted experiments in N2A cell lines overexpressing both CDK5/p35 and CDK5/p25. After subjecting the cells to two different concentrations, namely 50 nM and 100 nM, of each compound for a 24-h period, we observed a significant decrease in CDK5 activity, as illustrated in Figure 2E. This noteworthy reduction in CDK5 hyperactivity at the 100 nM concentration of BLINK11 and BLINK15 within the N2A cell model underscores the potential utility of these inhibitors in modulating CDK5 activity in a cellular environment. Also, we noticed a decreased activity for CDK5/p25 than CDK5/p35 in the presence of both BLINK11 and BLINK15 at 100 nM treatment, emphasizing their relevance for further exploration in addressing conditions associated with CDK5 dysregulation.

Our next step involved evaluating the ability of these compounds to cross the blood-brain barrier (BBB), a crucial consideration in drug targeting for the central nervous system. To address this challenge, we employed the parallel artificial membrane permeation assays (PAMPA) for BLINK11 and BLINK15. The results revealed that compounds BLINK11 and BLINK15 exhibited high permeability through the BBB, with P_aap_ values exceeding 10 × 10^-6^ (Figures S3A and S3B). This elevated permeability suggests that these compounds possess the capability to effectively traverse the BBB and access the central nervous system. This finding supports their candidacy for addressing CDK5 hyperactivity in neurodegenerative conditions associated with T2D. The ability of BLINK11 and BLINK15 to efficiently cross the BBB enhances their potential as therapeutic agents for targeting CDK5-related mechanisms in the central nervous system.

To assess the specificity of inhibitors BLINK11 and BLINK15 against CDK5, we investigated their effects on different Cdks, i.e., Cdk1, Cdk2, and Cdk9 that share structural homology with CDK5. Our findings indicate that Cdk1, in conjunction with its activator cyclin Y, exhibited inhibitory patterns with IC50 values of 39.34 nM for BLINK11 and 35.84 nM for BLINK15. However, these IC50 values were higher than those observed for CDK5/p35 and CDK5/p25. Conversely, Cdk2 (with cyclin A2) and Cdk9 (with cyclin T) showed minimal inhibition by both BLINK11 and BLINK15, with significantly higher IC50 values (Figures S3C–S3F). Overall, BLINK11 and BLINK15 can selectively target CDK5 at lower concentrations.

### LC-MS analysis reveals the bioavailability and distribution of BLINK11 and BLINK15 compounds in the HFD mouse model

**Figure 3:**
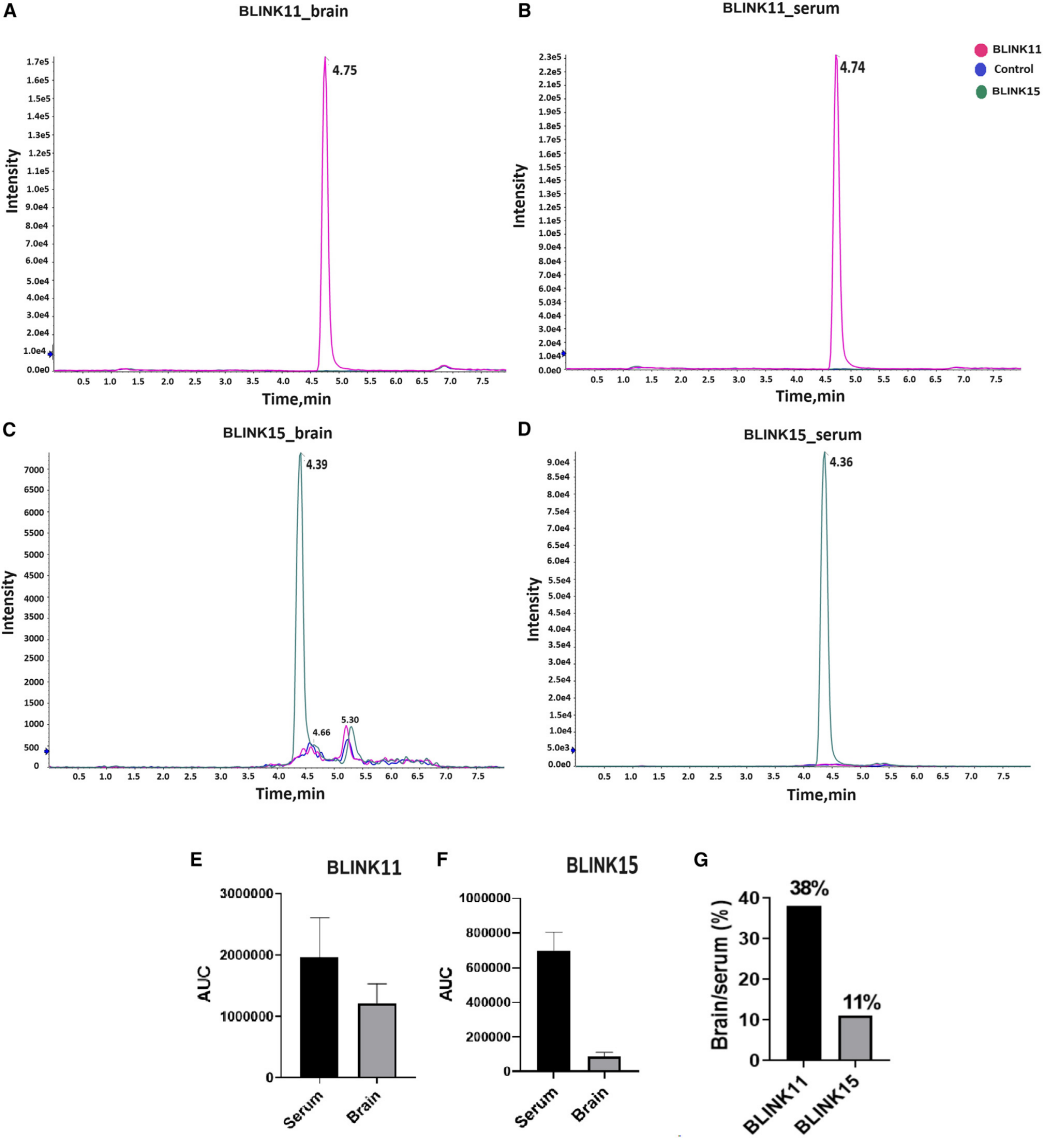
Bioavailability and distribution of compounds in brain lysate and serum (A and B) The graph shows the peaks of the BLINK11 compound in the (A) brain lysate and (B) serum. (C and D) The graph shows the peaks of the BLINK15 compound in the (C) brain lysate and (D) serum. (E and F) The bar graphs represent the area under the curve of the peaks of (E) BLINK11 in serum and brain (F) BLINK15 in serum and brain. (G) Percentages of BLINK11 and BLINK15 present in brain compared to serum is calculated and plotted in a bar graph.

To assess the BBB penetrability of BLINK11 and BLINK15 in the high-fat diet (HFD) mouse model, intraperitoneal injections were administered, and liquid chromatography-mass spectrometry (LC-MS) analysis was conducted on both brain lysate and serum samples (Figure 3). The LC-MS results depicted in Figures 3A and 3B showcase the peaks corresponding to the BLINK11 compound in the brain lysate and serum, respectively. Similarly, Figures 3C and 3D illustrate the peaks associated with the BLINK15 compound in the brain lysate and serum, respectively. The bar graph in Figure 3E represents the area under the curve of the peaks for BLINK11 in both serum and brain, while Figure 3F depicts the corresponding values for BLINK15 in serum and brain. These analyses provide a quantitative measure of the presence of the compounds in the respective samples. Furthermore, to ascertain the relative distribution of BLINK11 and BLINK15 in the brain compared to serum, the percentages of each compound present in the brain were calculated and plotted in a bar graph (Figure 3G). This assessment helps to elucidate the ability of these compounds to traverse the BBB and accumulate in the brain tissue. The LC-MS data presented in Figure 3 collectively contributes crucial insights into the bioavailability and distribution of compounds BLINK11 and BLINK15 in both the serum and brain lysate of the HFD mouse model. This information is pivotal for understanding the pharmacokinetics of the administered compounds and their potential impact on the central nervous system in the context of the studied neurodegenerative model.

### Effect of Cdk5 inhibitor treatment on body weight, glucose tolerance, insulin tolerance, and plasma insulin levels in rescued T2D mice

**Figure 4:**
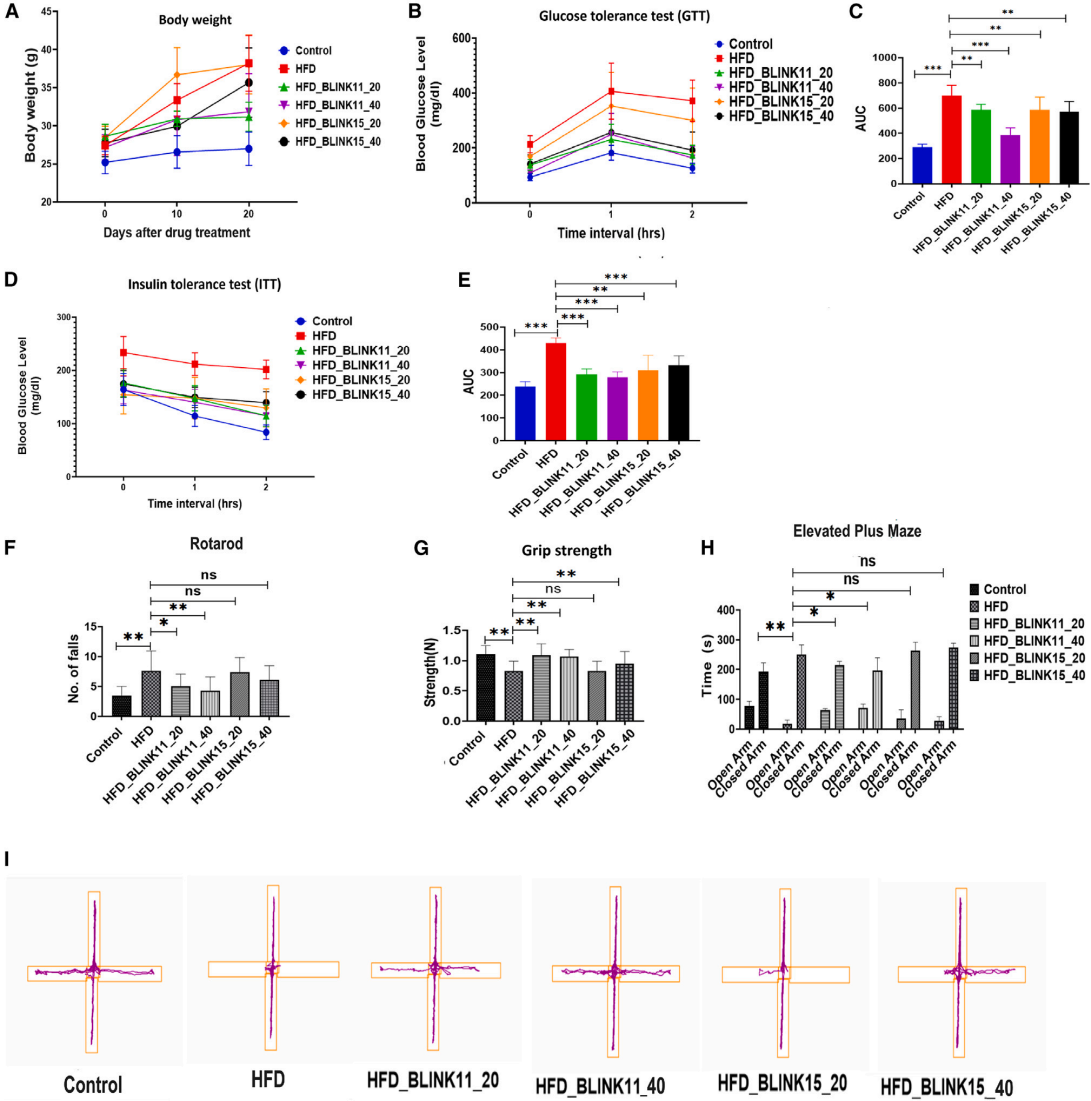
The establishment of a high-fat diet-induced diabetes mouse model with biochemical parameters is shown in graph (A) The graph showing body weights of control, HFD, HFD treated with BLINK11 20 mg/kg/day (HFD_BLINK11_20), HFD treated with BLINK11 40 mg/kg/day (HFD_BLINK11_40), HFD treated with BLINK15 20 mg/kg/day (HFD_BLINK15_20), and HFD treated with BLINK15 40 mg/kg/day (HFD_BLINK15_40) at 0, 10, and 20 days followed by drug treatment (*n* = 8 per group). (B) Glucose tolerant test (GTT). (C) Area under the curve (AUC) plots for GTT per group, one way ANOVA with Tukey’s test was applied to compare within the groups. (D) Insulin tolerant test (ITT). (E) Area under the curve (AUC) plots for ITT per group. Tukey’s multiple comparison test and one-way ANOVA was used for the analysis, and the values were provided as mean ± SD; ∗*p* < 0.05, ∗∗*p* < 0.01; ∗∗∗*p* < 0.001 and ns denotes non-significant. (F–H) Behavior of mice in diverse experimental paradigms. (F) Graph showing the number of falls in different mice groups while performing the rotarod test. (G) Grip strength measurements of the mouse groups. One-way ANOVA was performed for the analysis. (H) Elevated plus maze results show time spent by the mice in open-arm and closed-arm, two-way ANOVA with Tukey’s multiple comparison test were used for the analysis. (I) Representative track plots in EPM of different mice groups. All the experiments were performed with *n* = 8 per group and the values were provided as mean ± SD; ∗*p* < 0.05, ∗∗*p* < 0.01; ∗∗∗*p* < 0.001 and ns denotes non-significant.

The HFD-fed mice model received intraperitoneal injections of the compounds BLINK11 and BLINK15 at doses of 20 mg/kg/day and 40 mg/kg/day for a total of 20 days in the third month of HFD feeding. The body weight was measured at the beginning of the drug therapy, on day 0, and then again on days 10 and 20, as indicated in Figure 4A. The findings demonstrate a fast and steady increase in body weight in the 3-month-old HFD-induced mice group on days 10 and 20 compared to the control group following the administration of the drug (Figure 4A).

The BLINK11 drug-treated group is demonstrated to be rescued from the gain in body weight caused by a HFD for both dosages; however, for the BLINK15 drug-treated group, only the higher dose, i.e., 40 mg/kg/day, exhibits significant recovery (Figure 4A). Glucose tolerance test (GTT) and insulin tolerance test (ITT) were used to check the biochemical parameters for T2D in mouse models. Compared to the control group, the HFD group showed disturbed GTT and ITT, but the mouse group treated with BLINK11 and BLINK15 with both the dosages demonstrated a significant rescue in both GTT and ITT (Figures 4B–4E).

### Effectiveness of Cdk5 inhibitors BLINK11 and BLINK15 in alleviating cognitive impairment: Insights from behavioral experiments in HFD mouse models

#### Cdk5 inhibitor treatment rescues muscle strength and alleviates anxiety in a T2D mouse model

Following the assessment of the peripheral antidiabetic protective effects of the compounds BLINK11 and BLINK15, our investigation extended to evaluating a battery of behavioral tests commonly impaired in the context of T2D. Initially, we focused on examining muscle strength and coordination using the rotarod and grip strength tests. The rotarod test revealed notable findings in terms of forelimb muscular strength among the various experimental groups. Specifically, mice subjected to a HFD exhibited a significant decline in muscle strength compared to the control group. This observation suggests that a HFD may adversely impact an animal’s ability to build muscle and motor coordination. Encouragingly, mice from the HFD group treated with BLINK11 demonstrated a rescue effect, indicating a mitigation of the deterioration in muscle strength and motor coordination on the rotarod (Figure 4F).

In the grip strength test, the administration of compounds BLINK11 and BLINK15 at a dose of 40 mg/kg/day exhibited a rescue behavior, as illustrated in Figure 4G. This suggests that these specific treatments contributed to an improvement in grip strength compared to untreated counterparts. These findings indicate the potential therapeutic efficacy of BLINK11 and BLINK15 in addressing muscle strength deficits associated with T2D, emphasizing their role in mitigating the adverse effects of a HFD on musculoskeletal function.

Elevated anxiety levels have been identified as another notable concern in subjects with T2D. To investigate anxiety behaviors among the mice groups, we conducted an elevated plus maze (EPM) test, focusing on the time spent by each mouse in the open and closed arms of the apparatus. The results of the EPM test revealed distinctive patterns in anxiety-related behaviors. Mice subjected to a HFD displayed heightened anxiety compared to the control group, as evidenced by increased time spent in the closed arm of the maze (Figures 4H and 4I). Intriguingly, treatment with BLINK11 in the HFD group exhibited a rescuing effect on anxiety behavior, indicating a potential amelioration of anxiety levels associated with T2D. Contrastingly, the group treated with BLINK15 in the HFD showed no significant difference compared to the HFD group, as illustrated in Figures 4H and 4I. This suggests that while BLINK11 treatment demonstrates efficacy in alleviating anxiety behaviors, the effects may not be universally applicable across all compounds. These findings underscore the nuanced impact of specific treatments on anxiety-related behaviors in the context of T2D, warranting further exploration into the mechanisms underlying these observations.

#### Rescue of memory and spatial learning impairments in T2D mouse models with Cdk5 inhibitor treatment

**Figure 5:**
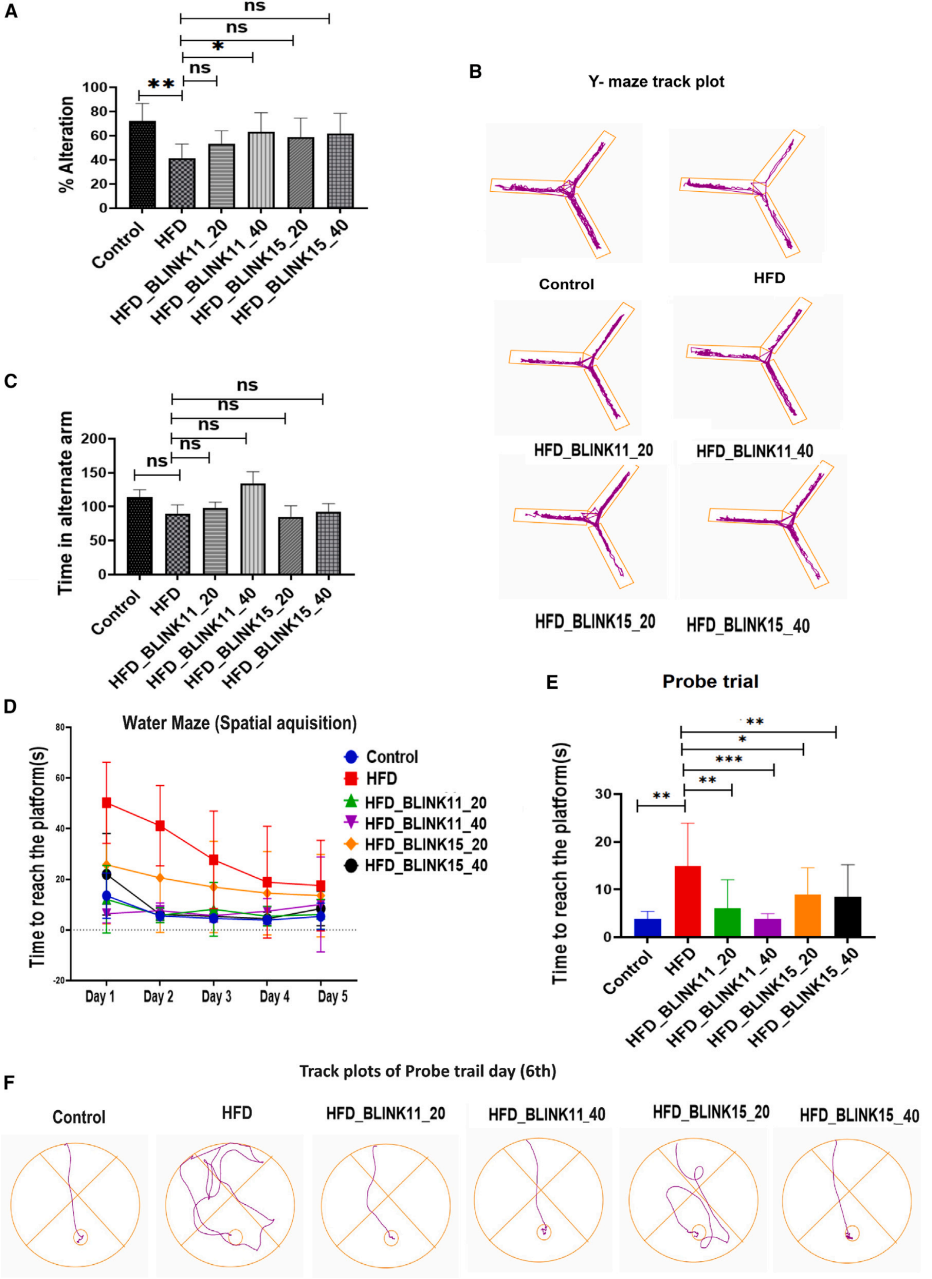
Behavioral study for memory and special learning through Y maze and Morri’s water maze (A) The graph represents the percentage alteration within the mice groups. (B) Control vs. HFD and HFD vs. BLINK11- and BLINK15-treated groups in Y maze. The images are the track plots of the mice groups in Y maze apparatus. (C) The graph showing time spent by the mice in altered arms of the Y maze apparatus. (D) The graph plots the time taken by the mice of different groups to reach the platform in the water maze during the training period from day 1 to day 5. (E) The bar graph shows the time taken by the mice per group to reach the platform in the probe trial day. Tukey’s multiple comparison test and one-way ANOVA were used for the analysis, and the values were provided as mean ± SD; ∗*p* < 0.05, ∗∗*p* < 0.01; ∗∗∗*p* < 0.001 and ns denotes non-significant. (F) The images represent track plot of water maze performed by the mice groups in the probe trial day. All the experiments were performed with *n* = 8 per group.

Memory and spatial learning are known to be impaired in the later stages of T2D. To investigate these impairments and explore potential drug treatments, we conducted Y-maze and water maze experiments on T2D mouse models. In the Y-maze, the alteration percentage of the HFD group was significantly lower than that of the control group, indicating memory and spatial learning deficits. Notably, mice in the HFD group treated with BLINK11 at a dose of 20 mg/kg/day did not exhibit any rescue effect, and similar results were observed with the BLINK15 compound treatment when compared to the untreated HFD group. However, in the HFD group treated with BLINK11 at a dose of 40 mg/kg/day, the decrease in alteration percentage was recovered. Additionally, the time spent in the altered arm showed no significant differences between the groups (Figures 5A and 5B).

Results from the water maze demonstrate a progressive learning pattern from the first to the fifth training days, with HFD mice models taking longer to reach the platform than the control group. In the HFD mouse treated with BLINK11 and BLINK15 at both doses, we can witness rescue behavior in this instance as well. According to the findings, the HFD mouse exhibits a reduction in spatial learning and memory. Treatment with BLINK11 and BLINK15 may be able to reverse this cognitive impairment brought on by a HFD and obesity. Compared to the BLINK15 treatment, the BLINK11 treatment in HFD exhibits a more remarkable improvement (Figures 5D–5F).

The results obtained from the water maze experiment unveiled a discernible pattern of progressive learning over the course of five training days. Notably, the HFD mouse models exhibited a prolonged duration to reach the platform compared to the control group, indicative of spatial learning challenges associated with HFD and obesity. However, in the HFD group treated with BLINK11 and BLINK15 at both doses, a noteworthy rescue behavior was observed. The cognitive impairment related to spatial learning and memory in HFD mice seemed to be mitigated with the administration of Cdk5 inhibitors BLINK11 and BLINK15. The data suggested that both compounds held the potential to reverse the detrimental effects induced by a HFD. Interestingly, when comparing the two treatments, the BLINK11 treatment in the HFD group exhibited a more remarkable improvement in spatial learning and memory, as illustrated in Figures 5D–5F. These findings underscore the therapeutic potential of Cdk5 inhibitors, particularly BLINK11, in ameliorating cognitive deficits associated with T2D conditions induced by an HFD, providing valuable insights for future research and potential clinical applications.

#### Enhanced Cdk5 activity and p25 generation in the HFD model rescued by Cdk5 inhibitor treatment

**Figure 6:**
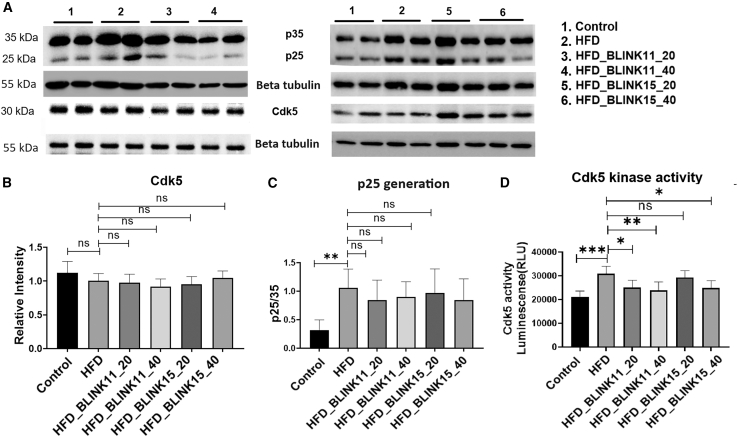
Cdk5 expression and activity along with p25 generation in treated vs. non treated HFD mouse groups (A) Western blot showing expression patterns of p35/25 and Cdk5 in the hippocampus region of mouse brain among the mouse groups control, HFD, and HFD treated group. (B) Densitometry of the respective blots Cdk5 with respect to the control beta-tubulin and (C) p25 with respect to p35 intensity. (D) Cdk5 kinase activity using pulled down Cdk5 from the hippocampus lysate of the mouse groups. All the datasets were analyzed using one-way ANOVA and Tukey’s multiple comparison tests, the data were provided as mean ± SD, *n* = 6. ∗∗*p* < 0.05, ∗∗*p* < 0.01, ∗∗∗*p* < 0.001 and ns denotes non-significant.

In our investigation of the molecular pathways contributing to cognitive impairments in the T2D mouse model, we have previously documented an association between elevated CDK5 activity and increased p25 generation in the brains of HFD mice, correlating with cognitive deficits.^23^ This study focuses on the hippocampal lysate of HFD mice, revealing consistent findings. The presence of p25 leads to heightened CDK5 activity in the brains of HFD mouse models. Notably, treatment with CDK5 inhibitors BLINK11 and BLINK15 results in a significant reduction in CDK5 activity, even in the presence of p25. Intriguingly, the hippocampal expression levels of CDK5 remain unaltered following treatment. Additionally, p25 generation in the BLINK11 and BLINK15 treated groups remains unchanged, as depicted in Figure 6.

#### Enhanced expression patterns of neurodegeneration markers in the HFD model are alleviated by the treatment of Cdk5 inhibitor BLINK11

**Figure 7:**
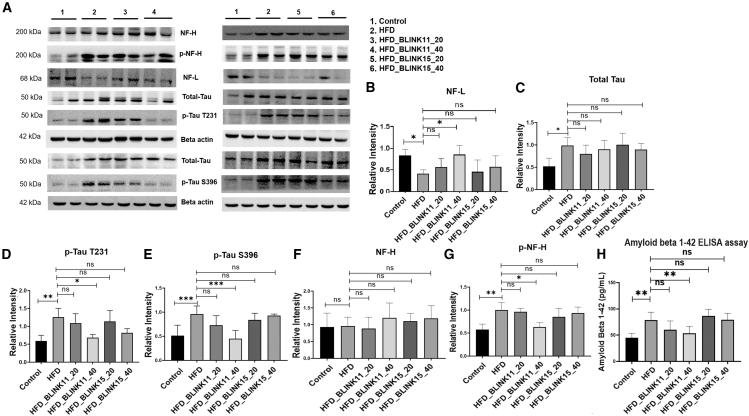
Expression patterns of neurodegenerative markers (A) The western blot images to check the expression pattern of NF-H, p-NF-H, NF-L, Tau, p-Tau-T231, and p-Tau-S396 in the hippocampus region of mouse brain among control, HFD non-treated, and HFD treated groups. (B–G) The densitometry analysis of the respect blots NF-L, total tau, Tau, p-Tau-T231, p-Tau-S396, NF-H, and p-NF-H. The ratio of the protein expression of p-tau and NF-H to the protein expression of total tau and NH-H was used to normalize the relative expressions. The expression levels of the rest of the proteins have been normalized with beta-actin. (H) Bar graph showing quantification of amyloid beta 1–42 in pg/ml in the mice groups using ELISA assay. Datasets were analyzed using one-way ANOVA and Tukey’s multiple comparison tests and were provided as mean ± SD, *n* = 6. ∗∗*p* < 0.05, ∗∗*p* < 0.01, ∗∗∗*p* < 0.001 and ns denotes non-significant.

The heightened cognitive deficits in the HFD mouse model prompted an exploration into neurodegenerative markers within the hippocampus. Despite unchanged neurofilament heavy chain (NF-H) levels, phospho-NF-H was notably increased in the HFD group compared to the control. The Cdk5 inhibitor BLINK11, particularly at a higher dose, significantly reduced phospho-NF-H levels. Additionally, neurofilament light chain (NF-L) levels, indicative of neurodegeneration, were diminished in the HFD group but restored with BLINK11 treatment. Tau and phospho-tau (231Thr and 396Ser) levels were substantially elevated in the HFD group, but BLINK11 treatment significantly decreased phospho-tau-396Ser levels (Figure 7).

**Figure 8:**
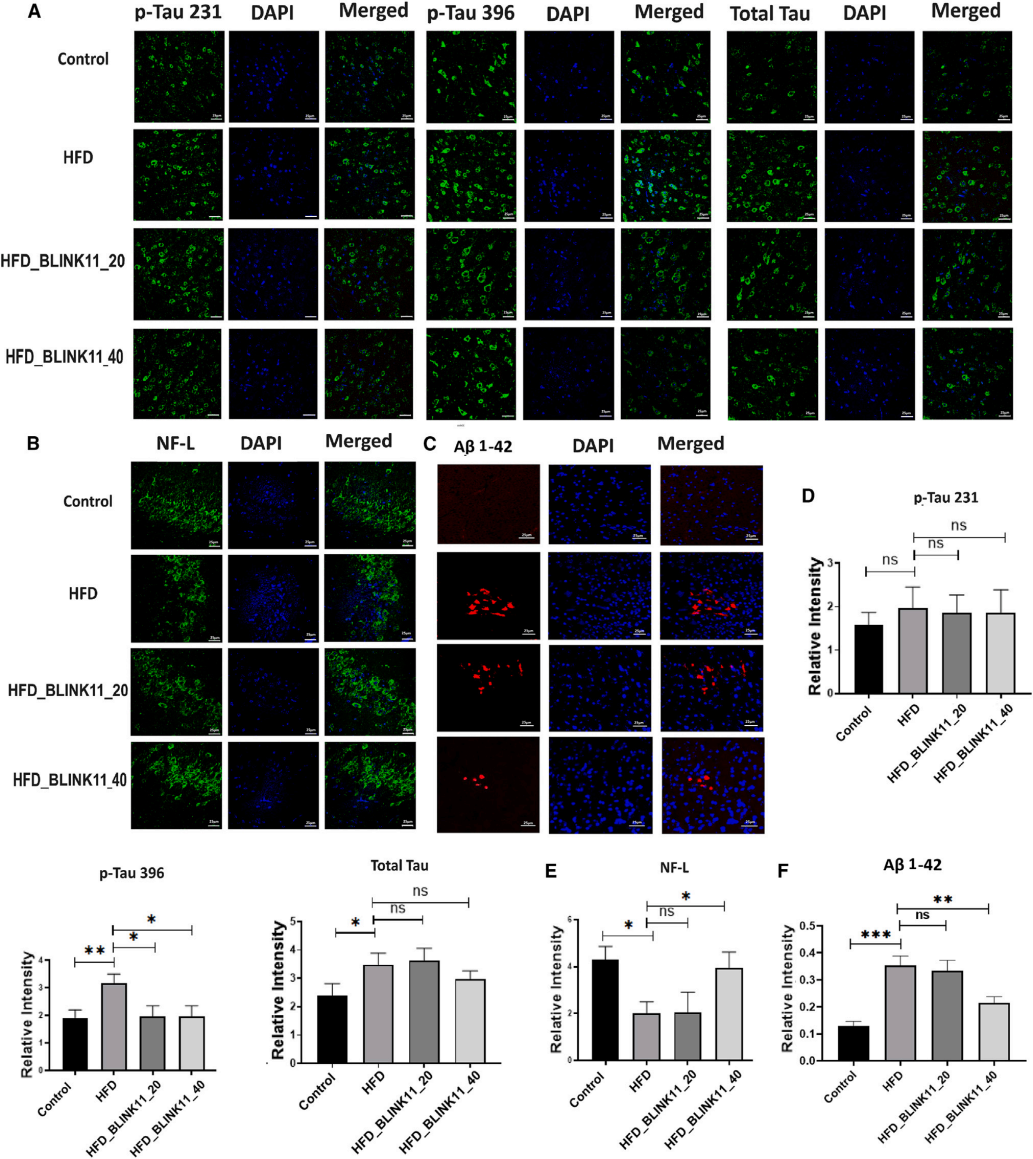
Immunohistochemistry results of neurodegenerative markers in mouse hippocampal brain sections (A) Immunofluorescence images of p-Tau T231, p-Tau S396, and total Tau expressions; (B) NF-L expressions; and (C) amyloid beta (Aβ) 1–42 expressions in the hippocampus region of the brain sections sliced with 10-micron thickness. (D–F) Bar graph showing the intensity of the respective proteins (D) p-Tau T231, p-Tau S396, and total Tau; (E) NF-L; and (F) Aβ 1–42 with respect to their corresponding DAPI intensities. All the datasets were analyzed using one-way ANOVA with Tukey’s multiple comparison tests, the data were provided as mean ± SD, *n* = 6. ∗∗*p* < 0.05, ∗∗*p* < 0.01, ∗∗∗*p* < 0.001 and ns denotes non-significant.

Immunohistochemistry on hippocampal sections confirmed these findings, showing consistent expression patterns of NF-L, p-Tau 231Thr, p-tau 396Ser, and total Tau with the western blot results (Figure 8). These results suggest that Cdk5 inhibition by BLINK11 mitigates HFD-induced neurodegeneration through the inhibition of phosphorylation events involving NF-H and tau proteins. Moreover, ELISA assay as well immunohistochemistry staining with amyloid beta (Aβ) 1–42 revealed a significant increase in Aβ 1–42 accumulation in the hippocampus of the HFD group, which was significantly reduced by BLINK11 at 40 mg/kg treatment (Figures 7H, 8C, and 8F). Thus, BLINK11 at this dosage effectively rescues neurodegeneration caused by HFD.

## Discussion

The global rise in the prevalence of T2D and AD is a growing concern, emphasizing the need for a deeper understanding of their pathophysiology.^24^^,^^25^ The strong evidence linking these two conditions underscores the importance of exploring shared mechanisms that contribute to their progression. Chronic glucose toxicity has been identified as a major contributor to neurological impairments associated with diabetes, providing a foundation for investigating potential therapeutic interventions.^26^^,^^27^^,^^28^ CDK5 hyperactivation has emerged as a key player in the progression of neurodegeneration, neuroinflammation, and cognitive impairment in various neurological disorders.^13^^,^^29^^,^^30^^,^^31^^,^^32^ Neurofilament tangles (NFTs) production, protein aggregation, tau-phosphorylation, and neuroinflammation are influenced by dysregulation of CDK5.^8^^,^^33^ Multiple studies, including the present one, have demonstrated that inhibiting CDK5 can significantly improve neurodegenerative phenotypes.^34^^,^^35^^,^^36^^,^^37^ The study builds on prior research indicating that high glucose exposure leads to CDK5 hyperactivity and oxidative stress, resulting in hyperphosphorylation of tau.^19^^,^^30^^,^^38^ Importantly, the current investigation extends these findings to suggest that CDK5 hyperactivation under glucotoxicity may contribute to the loss of beta cell function and insulin resistance, proposing CDK5 inhibitors as potential candidates for the treatment of T2D.

Previous research, including our work, has highlighted the hyperactivation of CDK5 and the generation of p25 in HFD mouse models of T2D, coinciding with cognitive decline.^23^^,^^38^^,^^39^ This study takes a significant step forward by screening a library of CDK5 inhibitors for their potential to rescue T2D phenotypes, cognitive impairments, and neurodegeneration in a T2D mouse model induced by a HFD.

The initial phase of the study involved a comprehensive *in-silico* screening of the KINACore library for CDK5 inhibitors using molecular docking. The library was meticulously designed based on structural similarities with known kinase activators and the adenosine portion of ATP. The identification of 50 promising compounds through HTVS laid the groundwork for further *in vitro* evaluations. The top eight compounds with low IC50 values were selected for additional screening, considering both their efficacy and low cytotoxicity across multiple cell lines (Figures 1 and 2).

To assess the BBB penetration and bioavailability of the selected compounds, LC-MS analysis was conducted on brain lysate and serum samples (Figure 3). The results indicated distinct peaks corresponding to the compounds BLINK11 and BLINK15, emphasizing their potential to reach the central nervous system. Subsequent evaluation of biochemical parameters relevant to T2D phenotypes in HFD mice treated with BLINK11 and BLINK15 revealed a rescue in weight gain, improved glucose tolerance, and reduced insulin levels compared to the untreated HFD group. These findings support the potential of CDK5 inhibitors in mitigating peripheral complications associated with T2D (Figure 4).

Moving beyond peripheral effects, the study explored the impact of CDK5 inhibitors on behavioral and cognitive aspects in the HFD mouse model. The assessment of muscle strength and coordination through the rotarod and grip strength tests demonstrated notable deficits in the HFD group, while treatment with BLINK11 exhibited a rescuing effect. Additionally, both BLINK11 and BLINK15 treatments showed a rescue behavior in the grip strength test. Elevated anxiety levels, commonly associated with T2D, were assessed using the EPM test. BLINK11 treatment exhibited a rescuing effect on anxiety behavior, highlighting its potential in ameliorating anxiety levels associated with T2D (Figures 4F–4I).

Cognitive impairments, a significant concern in both T2D and neurodegenerative diseases, were evaluated through Y-maze and water maze experiments. The results indicated significant deficits in memory and spatial learning in the HFD group, with BLINK11 treatment showing a potential cognitive benefit. Both BLINK11 and BLINK15 treatments demonstrated a rescue behavior in the water maze, with BLINK11 exhibiting a more remarkable improvement in spatial learning and memory (Figure 5).

The study delves into the molecular pathways underlying cognitive impairments in the T2D mouse model induced by an HFD. Elevated CDK5 activity and increased p25 generation in the brains of HFD mice were consistent with prior findings, and treatment with CDK5 inhibitors, particularly BLINK11, resulted in a significant reduction in CDK5 activity. Importantly, BLINK11 treatment effectively reversed elevated phospho-NF-H levels and restored diminished expressions of NF-L,^40^^,^^41^ indicating a potential neuroprotective effect. Tau hyperphosphorylation, a hallmark of neurodegeneration, was substantially reduced with BLINK11 treatment, further supporting its therapeutic potential. Immunohistochemistry results reinforced these findings, providing a comprehensive understanding of the impact of CDK5 inhibition on neurodegenerative markers (Figures 7A–7G).

Quantification of Aβ1–42 in the brain lysate and immunohistochemistry in the brain section revealed a significant increase in Aβ1–42 accumulations in the HFD group, with BLINK11 treatment showing a significant rescue effect (Figures 7H, 8C, and 8F). These results collectively underscore the therapeutic efficacy of CDK5 inhibition, particularly with the compound BLINK11, in alleviating neurodegeneration induced by an HFD in mouse models. The modulation of neurodegenerative markers, including NF-H, NF-L, and tau phosphorylation, highlights the potential of CDK5 inhibitors as promising therapeutic interventions for neurodegenerative conditions associated with metabolic disorders.

In conclusion, this study provides a comprehensive investigation into the potential of Cdk5 inhibitors, particularly BLINK11, in addressing both peripheral and cognitive complications associated with T2D induced by an HFD. The multi-faceted approach, combining *in silico* screening, *in vitro* evaluations, and *in vivo* assessments, strengthens the validity of the findings. The demonstrated rescue effects on muscle strength, anxiety levels, and cognitive impairments underscore the therapeutic potential of Cdk5 inhibitors, offering promising avenues for further research and clinical applications. The study not only contributes to our understanding of the molecular mechanisms underlying T2D-associated neurodegeneration but also positions Cdk5 inhibition as a compelling strategy for mitigating the complex interplay between metabolic disorders and cognitive decline.

### Limitations of the study

While our study demonstrates promising neuroprotective and antidiabetic effects of Cdk5 inhibitor, BLINK11, over a 20-day treatment period, it does not fully capture the chronic and progressive nature of T2D-associated neurodegeneration. Long-term studies are essential to assess sustained efficacy, potential adaptive resistance, and safety concerns, including metabolic consequences. Future research should also explore whether prolonged Cdk5 inhibition maintains its protective effects.

## Resource availability

### Lead contact

For further information or requests for resources and reagents, please contact the lead author, Dr. Binukumar Balachandran Krishnamma (binukumar@igib.in, binukumarbk@gmail.com).

### Materials availability

This study did not produce any unique reagents.

### Data and code availability

• The lead contact will provide all reported data upon request.
• This paper does not include original code.

## Acknowledgments

This research received financial backing from 10.13039/501100001412CSIR India, specifically through grants OLP2301 and MLP2008, as well as support from the 10.13039/501100001843Science and Engineering Research Board India, through grant GAP0168. CSIR provided senior research fellowships for S.P. and J.B. R.C. acknowledges DBT-RA and CMNKPDF fellowships. The funders played no role in the decision to publish or in the manuscript’s creation. Special thanks go to Mukesh Kumar for his valuable assistance in performing the mice euthanizing procedure during the animal experiments. We express our gratitude to the IGIB Mass Spectrometry Facility run by Shantanu Sengupta lab for their valuable assistance in quantifying the kinase inhibitors in both brain and plasma samples. We extend our sincere gratitude to Dr. Kam Y.J. Zhang, Team Leader of the Laboratory for Structural Bioinformatics at the Center for Biosystems Dynamics Research, RIKEN, Japan, for generously providing computational resources and engaging in insightful discussions.

## Author contributions

B.B.K. conceived the presented idea. S.P. carried out the *in vitro* and *in vivo* experiments. D.K.V. and R.C. performed all the molecular modeling studies. S.P., D.K.V., R.C., and B.B.K. wrote the manuscript. J.B. made the graphical abstract. P.Singh performed the mass spectroscopy experiments. P.Shetty., S.C., and S.M. collaborated in synthesizing the BLINK11 compound. S.P., J.B., and B.B.K. contributed to the final version of the manuscript.

## Declaration of interests

The authors declare no competing interests.

## STAR★Methods

### Key resources table

**Table:** 

REAGENT or RESOURCES	SOURCE	IDENTIFIER
**Antibodies**		

Cdk5	CST	Cat# 2506; RRID: AB_2078855
p35/25	CST	Cat#C64B10; RRID: AB_1078214
Neurofilament L	Invitrogen	Cat#4747-RBM3-P1ABX; RRID: AB_2532995
Neurofilament H	Invitrogen	Cat#BS-0708R; RRID: AB_1077155
Tau	Affinity Biosciences	Cat#AF2719; RRID: AB_2847771
p-Tau T-231	Invitrogen	Cat#710126; RRID: AB_2532360
p-Tau S396	Invitrogen	Cat#44-752G; RRID: AB_2533745
p-NF H	Invitrogen	Cat#4744-MSM3-P0; RRID: AB_1077155
GFAP	Abcam	Cat#13–0300; RRID: AB_2532994
Beta-actin	Sigma Aldrich	Cat#A5441; RRID:AB_476744
Beta tubulin	Invitrogen	Cat#480011; RRID:AB_2532242
Alexa Flour Rabbit 488 nm	Thermofisher	Cat#A-11008; RRID:AB_143165
Alexa Flour Mouse 488 nm	Thermofisher	Cat#A28175; RRID:AB_2536161
Alexa Flour Rabbit 594 nm	Thermofisher	Cat#A-11012; RRID: AB_2534079
Alexa Flour Mouse 594 nm	Thermofisher	Cat#A-11032; RRID: AB_2534091

**Reagents**		

DMEM-high glucose powder	Gibco	12100046
Fetal Bovine Serum	Gibco	16000044
Sodium Bicarbonate	Sigma Aldrich	W700433
Lipofactemine-3000	Invitrogen	L3000015
MTT (3-(4,5-Dimethylthiazol-2-yl)-2,5-Diphenyltetrazolium Bromide)	Invitrogen	M6494
DMSO (Dimethyl sulfoxide)	Merck	67-68-5
Tween 20	CDH	9005-64-5
Triton X-100	CDH	9002-93-1
D-Glucose	Sigma Aldrich	G8270
Insulin	Sigma Aldrich	I6634
Immobilized Protein A/G Resin	GBioscience	786–837
Brain Polar Extract	Avanti	141101P
PAMPA filter plates	Sigma Aldrich	MAIPNTR10
40% Formaldehyde	Fisher Scientific	24008
Methanol	ThermoFisher Scientific	ALF-022909-K2

**Commercial assays**		

ADP-Glo Kinase assay kit	Promega	V6930

**Software and algorithms**		

GraphPad Prism 8.4.3	GraphPad	https://www.graphpad.com/updates/prism-843-release-notes
ImageJ Fiji	GitHub	https://downloads.imagej.net/fiji/latest/fiji-win32.zip
ANY-Maze	ANY-Maze	https://www.any-maze.com/support/downloads/
Glide	Schrödinger Maestro 11.8	https://www.schrodinger.com/
Desmond	Desmond for academics 2022-4	https://www.deshawresearch.com/index.html

### Experimental model and study participant details

#### Cell culture

HEK293T, HepG2, and N2A cells were used in the study representing kidney, liver and neuronal cell lines respectively. The cells were cultured in DMEM supplemented with 10% fetal bovine serum and antibiotics at 37°C under 5% CO2. The cells were tested negative for mycoplasma.

#### Animal model

Male C57BL/6J mice were used in this study and assigned to two dietary groups: a control group receiving a standard diet with 10% fat and a HFD group fed a diet containing 60% fat to induce obesity. The dietary intervention was maintained for 20 weeks. Mice were housed under controlled conditions at 25°C with a 12-h light/dark cycle. All animal experiments were conducted in strict accordance with protocols approved by the Institutional Animal Care and Use Committee (IAEC) of CSIR-IGIB (IAEC Approval Number: IGIB/IAEC/21/29, IGIB/IAEC/JUL2019/17).

### Method details

#### Screening of Cyclin-dependent kinase 5 inhibitors from KINACore

The KINACore library, containing over 20,000 drug-like molecules from ChemBridge, was screened for Cdk5 inhibitors. The library was prepared using the LigPrep module of Schrödinger (Schrödinger, LLC, New York, USA), generating different tautomeric and ionization states via the Epik program, followed by energy minimization using the OPLS-2005 force field. These prepared ligands were then subjected to HTVS against Cdk5.^42^^,^^43^

#### Selection, preparation of Cyclin-dependent kinase 5 structure, and high-throughput virtual screening

Structural analysis of Cdk5 from PDB revealed conformational changes in amino acids upon ligand binding. Four distinct receptor conformations were selected for screening. These structures were prepared using Schrödinger Maestro’s Protein Preparation Wizard, with steps including the addition of polar hydrogen atoms, protonation states optimization and hydrogen bond network optimization. Energy minimization was performed with an OPLS-2005 force field. A grid around the bound ligands’ centroid was generated for docking studies. The virtual screening protocol involved HTVS, SP, and XP dockings, selecting the top 30% of compounds at each step for MM-GBSA calculations using the Schrödinger Prime module.

#### Molecular dynamic simulation

To further observe the stability of the Cdk5-ligand complexes for a period of time, we performed MD simulations (MD-Simulations) using Desmond software with GPU acceleration.^44^ The Cdk5-ligand complexes with top binding energies were selected for MD-simulations. Using the System Builder module, we prepared the protein-ligand complex for MD simulation by keeping them in an orthorhombic simulation box with a dimension of 5 × 5 × 5 Å containing TIP3P water molecules. To neutralize the system, a required number of Na+ or Cl-ions was added. MD simulation was performed using an OPLS-2005 force field for 100 ns, with a recording interval of 100 ps. We utilized the NPT ensemble, maintaining constant temperature (Fixed at 300K) and pressure (fixed at 1.01 bar). Temperature and pressure control were implemented through the Nose-Hoover chain and Martyna-Tobias-Klein methods, respectively. Integration was performed by utilizing the RESPA algorithm with a time step of 2 fs and default configurations were applied to all other simulation parameters.

#### Compound synthesis

The 19 compounds screened through molecular docking are procured from Chembridge for initial *in vitro* screening. The bulk synthesis was done for *in vivo* experiments. The synthesis steps for BLINK11 are as follows:

Reactions were set-up on fume hoods and carried out under nitrogen atmosphere in Schlenk tubes unless otherwise noted. Compounds were purified by flash chromatography (Isolera-Biotage) using silica gel (200–400 mesh). All the reagents and solvents were used as received from commercial sources, unless otherwise specified. 1H NMR data were recorded on Bruker 400MHz AVANCE series or Bruker300 MHz DPX Spectrometer with CDCl3 or DMSO-d6 as solvent. 1H chemical shifts were referenced to CDCl3 at 7.26 ppm and for DMSO-d6 at 2.51 and 3.20 ppm. Multiplicities are abbreviated as follows: singlet (s), doublet (d), triplet (t), quartet (q), doublet-doublet (dd), quintet (quint), sextet (sextet), septet (septet), multiplet (m), and broad (br). MS was measured on Agilent 7890A/5975C Series GC/MSD mass spectrometer or Agilent 1100 Series LC/MSD mass spectrometer.

The synthesis of target compound 7 for biological evaluation involves the above synthetic sequence depicted in Scheme 1. The synthesis of intermediate 4 was first accomplished via 3 steps. The first step involves the nucleophilic displacement of 3,4 dichloro nitro benzene with morpholine, followed by reduction and subsequent reaction with chloro acetyl chloride to yield compound 4. The synthesis of compound 6 involves the amide formation reaction of compound 5 and 2,3 dichloro aniline. The intermediates 4 and 6 synthesized were then coupled under basic conditions to deliver the target compound 7.

**Figure:**
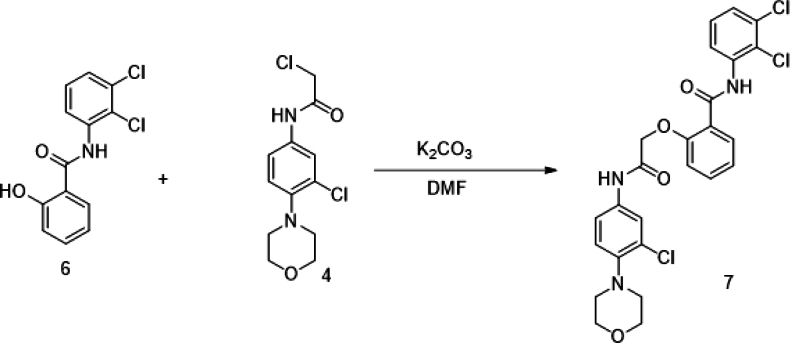

Synthesis of 2-chloro-N-(3-chloro-4-morpholinophenyl)acetamide (4)

To a stirred solution of 3-chloro-4-morpholinoaniline (3) (1.0 g) in DCM (20 mL) was added TEA (1.5 eq.) followed by chloroacetylchloride (1.1 eq.) at 0°C and 5°C. The reaction mixture was gradually warmed to RT and stirred at RT for 4h. The reaction was monitored by LCMS. After completion of reaction was quenched with ice-cold water (30 mL) and extracted with DCM (2 x 30 mL). The combined organic layers were dried over sodium sulfate and concentrated over vacuo to afford crude was purified by flash chromatography (40% EA:Hexane) to afford 2-chloro-N-(3-chloro-4-morpholinophenyl)acetamide (4) (1.2g) as brown solid. LCMS (M + H): 289.

**Figure:**
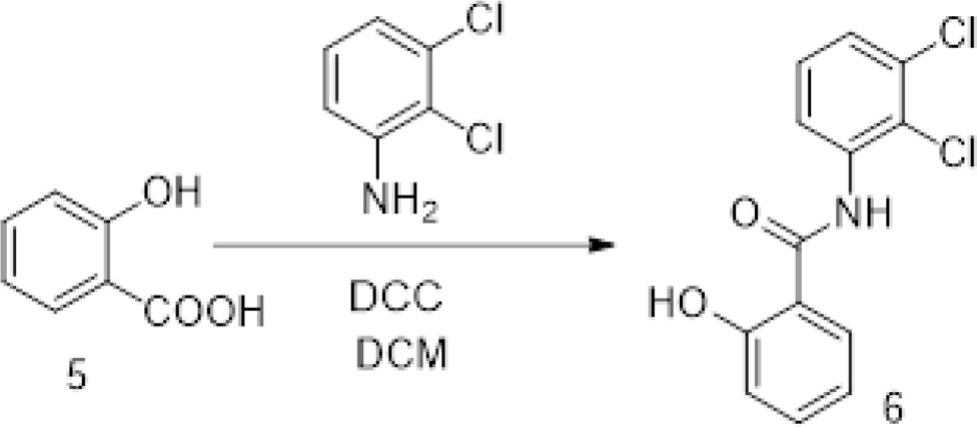

Synthesis of N-(2,3-dichlorophenyl)-2-hydroxybenzamide (6)

To a stirred solution of salicylic acid (5) (1g) and 2,3-dichloroaniline (1 eq.) in DCM (20 mL) was added (1-(3-dimethylaminopropyl)-3-ethylcarbodiimide) (1.5 eq.). The reaction mixture was heated to reflux for overnight. The reaction was monitored by LCMS. After completion of reaction the reaction mixture was concentrated under vacuum, the crude was purified by flash coloumn chromatography (30–80% EA:Hexane) to afford N-(2,3-dichlorophenyl)-2-hydroxybenzamide (6) (500 mg) white solid. LCMS (M + H): 282.

**Figure:**
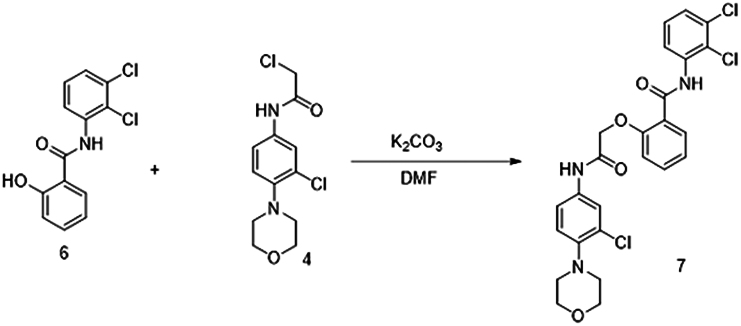

Synthesis of 2-(2-((3-chloro-4-morpholinophenyl)amino)-2-oxoethoxy)-N-(2,3-dichlorophenyl)benzamide (7)

To a stirred solution of N-(2,3-dichlorophenyl)-2-hydroxybenzamide (6) (500 mg) in DMF (10 mL) was added anhydrous K2CO3 (2 eq.) followed by 2-chloro-N-(3-chloro-4-morpholinophenyl) acetamide (4) (1.1eq.). The reaction mixture was heated to 80°C for 16 h. The reaction was monitored by LCMS. After completion of reaction, the reaction mixture was quenched with ice-cold water (30 mL) and extracted with ethyl acetate (2 x 30 mL). The combined organic layers were dried over sodium sulfate and concentrated over vacuo to afford crude was purified by flash chromatography (3% MeOH:CHCl3) to afford 2-(2-((3-chloro-4-morpholinophenyl)amino)-2-oxoethoxy)-N-(2,3-dichlorophenyl)benzamide (7) (400 mg) as off white solid.

LCMS (M + H): 534. 1H NMR (400 MHz, DMSO-d6) δ 10.66 (s, 1H), 10.41 (s, 1H), 8.2 (d, J = 8 Hz, 1H), 7.9 (d, J = 8 Hz, 1H), 7.7–7.72 (m, 1H), 7.58–7.63 (m, 1H), 7.38–7.47 (m, 3H), 7.1–7.24 (m, 3H), 5.1 (s, 2H), 3.66–3.74 (m, 4H), 3.86–3.93 (m, 4H).

BLINK15 was synthesized from leap chem co. ltd. The synthesis of BLINK15 with smiles COc1ccc(cc1N+ = O)C(=O)C[C@@]2(O)C(=O)N(c(*c*23)ccc(Br)c3)Cc4ccccc4 begins with 4-nitroanisole (COc1ccc(cc1N+ = O)) as the starting material. First, introduce the formyl group at the para position relative to the methoxy group using a formylation reaction, such as the Vilsmeier-Haack reaction, to obtain 4-nitro-2-methoxybenzaldehyde (COc1ccc(cc1N+ = O)C(=O)H). Next, perform an Aldol condensation to react the formylated product with an appropriate enolate, forming the α,β-unsaturated ketone intermediate (COc1ccc(cc1N+ = O)C(=O)CH = CH2). Proceed with a Michael addition using a suitable nucleophile to introduce the side chain, preparing the molecule for further cyclization. Then, use intramolecular cyclization to form the bicyclic lactam structure, creating the C2-*O*-C3 bond and resulting in the fused ring system (C[C@@]2(O)C(=O)N(c(*c*23)ccc(Br)c3)). Introduce the bromine atom through an electrophilic aromatic substitution reaction with bromine (Br2) in the presence of a catalyst, obtaining the bromo-substituted aromatic ring.

#### MTT assay and transfection

To evaluate the compound toxicity for the Cdk5 inhibitors, an MTT assay was performed in HEK293T, HepG2, and N2A cells at varying concentrations. Six hours after cell seeding, the compounds were added in different amounts, and the cells were harvested after 24 h for further analysis. N2A cells were transfected using Lipofectamine-3000 reagent, co-transfecting with plasmids encoding Cdk5/p35 and Cdk5/p25 for 12 h, followed by inhibitor treatment to assess their effects in the cell culture system. The concentrations of each plasmids used was 1 μg, total of 3 μg per well for 6 well plates.

#### Drug administration in mice model

Two groups of four-week-old male C57BL/6J mice were established. One group (*n* = 8) was fed a standard laboratory mouse chow diet (Protein: 20% kcal, Carbohydrate: 70% kcal, Fat: 10% kcal), while the other group (*n* = 40) was placed on a HFD (Protein: 20% kcal, Carbohydrate: 20% kcal, Fat: 60% kcal).^23^ Body weight, ITT, and GTT were assessed. Mice were treated with BLINK11 and BLINK15 inhibitors at 20 mg/kg and 40 mg/kg doses for 20 days. The study included six groups: Control, HFD, HFD_BLINK11_20, HFD_BLINK11_40, HFD_BLINK15_20, and HFD_BLINK15_40. Figure S4 shows the flow chart of the HFD model generation and drug treatments. The control used in the study are vehicle treated-control group (5% DMSO, 0.1% tween 20 and 1X PBS). The doses of 20 mg/kg and 40 mg/kg body weight were selected based on evidence from previous studies evaluating the efficacy and safety of similar kinase inhibitors.^45^^,^^46^^,^^47^ The 20 mg/kg dose was chosen as a lower effective dose, supported by its ability to achieve therapeutic plasma concentrations without adverse effects in comparable models. The 40 mg/kg dose was selected to assess dose-dependent efficacy and represents the upper limit of tolerability based on pharmacokinetic and toxicity data. These doses were also informed by preliminary experiments in our laboratory, which demonstrated significant pharmacological activity within this range, ensuring a balance between efficacy and safety.

#### GTT and ITT assay

For the glucose tolerance test, 2 g/kg of glucose was utilized, and 0.75U/kg of human insulin was employed The glucose tolerance test (GTT) and insulin tolerance test (ITT) were performed in HFD mice models after 20 weeks of HFD feeding followed by 20 days of drug administration.^48^ For GTT the mice were kept in a fasting period for overnight before glucose injections of 2 g/kg intraperitoneally. For ITT the mice were kept in a fasting for 6 h prior to insulin injection of 0.75 U/kg intraperitoneally. A one-touch glucometer was used to assess the blood sugar levels at various intervals (0, 60, and 120 min) following intraperitoneal infusion of both glucose and insulin.^23^^,^^49^^,^^50^^,^^51^ After 20 weeks of a HFD and 20 days of drug administration, the tests were carried out.

#### Kinase assays and immunoprecipitation

The test tube Cdk5 Kinase assay was performed utilizing Cdk5/p35 and Cdk5/p25 complexes as kinases, following the modified procedure outlined in a previous study,^52^ using a Promega ADP-GloTM Kinase Assay kit. The reaction was carried out with 10 μM ATP and 2 μg of histone as the substrate for Cdk5 complexes. To study the inhibition patterns, 19 Cdk5 inhibitors (BLINK1-19) identified through molecular docking were added to the Cdk5 kinase reaction in increasing concentrations ranging from 0 to 100 nM. The IC50 (half-maximal inhibitory concentration) values of all the inhibitors were calculated based on Log(inhibitor) vs. normalized response using GraphPad software.

Cdk5 was immunoprecipitated from N2A cells and brain lysate using a Cdk5 antibody. Kinase assays were performed using a Promega ADP-Glo Kinase Assay kit with Cdk5/p35 and Cdk5/p25 complexes. Histone was used as a substrate to test kinase activity Cdk5 was immunoprecipitated from N2a cells overexpressing Cdk5/p35 and Cdk5/p25, following treatment with BLINK11 and BLINK15 inhibitors at concentrations of 50nM and 100nM, using a Cdk5 antibody. A kinase assay was subsequently conducted as described in Bankston et al.^53^ Additionally, Cdk5 was precipitated from mouse brain lysates treated with BLINK11 and BLINK15 inhibitors. The immunoprecipitation was performed with 100 μL of A/G beads, 2 μg of Cdk5 antibody, and 150 μg of cell and brain lysates, with elution carried out using a urea elution buffer. The kinase assay was conducted using the ADP-Glo Kinase Assay Kit (Promega) with immunoprecipitated Cdk5, ATP, and 1–2 μg of histone as the substrate.

#### Behavioral experiments

The darkest time of the housing state was used for all behavioral studies. An hour before the experiment, the mice were acclimated in the behavior setup room. The Anymaze software (ANY-maze Video Tracking Software from Stoelting Co, 620 Wheat Lane Wood Dale, IL 60191 USA) was used for video tracking and data collection.

##### Grip strength

A grip strength meter was used to measure the forelimb grip strength in mice.^54^ The mouse was given a chance to grab the gauge with its forelimbs before having its tail gently pulled back. Digital transducers were used to record the muscular force. Five trials were conducted on each mouse.^23^

##### Rotarod

Rats and mice have been tested for motor coordination using the rotarod test. The animals were placed on a horizontal rod that rotates around its long axis for this test; in order to remain upright and prevent sliding off, the animal must move ahead. Every 5 min, the rod’s speed was raised, and the number of falls were counted to assess the motor coordination.^23^^,^^55^

##### EPM

In order to examine anxiety-like behavior, phenotype transgenic and knockout mice, and screen for anxiolytic and anxiogenic medicines, this maze takes advantage of the natural fear of open, high areas in rodents.^56^^,^^57^ Each animal had a 10-min test, during which we measured the entry into the open and closed arms as well as the time and distance spent in each component.

The EPM experiment is a widely utilized technique in behavioral neuroscience to evaluate anxiety-related behaviors in mice.^57^ The apparatus comprises two open arms and two closed arms arranged in a plus-shaped configuration and elevated above the ground. Mice are introduced to the center of the maze, and their movements are observed over a set period of 5 min.^56^^,^^58^ The amount of time spent in the open arms versus the closed arms is recorded, with more time in the open arms indicating lower anxiety levels. The distance traveled in each arm were also analyzed using ANYmaze software. This method leverages the natural tendency of rodents to avoid open spaces and seek enclosed areas.

##### Y-maze test

As previously mentioned, the Y-maze test was utilized to evaluate the mouse groups' short-term spatial memory The Y-maze test was used to assess short-term spatial memory in the mouse groups. The apparatus consists of three arms arranged in a Y-shape, typically labeled A, B, and C, with each arm having the same length and width. Mice were initially placed in one arm (usually arm A) and allowed to explore freely for 5 min, with one of the other arms blocked off, restricting exploration to the starting arm and the remaining open arm. After a 5-min delay, the mouse was reintroduced to the maze with all arms accessible. The time spent in each arm and the number of entries into each arm were recorded over a 5-min period. A higher number of entries into the previously blocked arm indicates improved spatial memory and cognitive function, suggesting the mouse recognized the novel arm. We also evaluated the mice’s spontaneous alternation rate during the Y-maze test. Spontaneous alternation reflects working memory, as it refers to the mouse’s tendency to enter a less recently visited arm. The sequence and number of arm entries were tracked, with an alternation defined as consecutive entries into all three arms without repetition (e.g., A-B-C, B-C-A).^59^^,^^60^^,^^61^ During the training phase, one arm (identified as the new arm) was blocked in an indistinct, Y-shaped area that made up the maze. During the training phase, a single mouse was set free at the lower arm & given 5 min to explore the maze. The mice underwent the test phase for 5 min apiece following an hour-long intertrial break, during which time the previously blocked arm (new arm) was now available. As a result, we evaluated the novel arm’s exploration according to many criteria.

Spontaneous Alternation (%) = {(Number of alternations)/ (Total arm entries−2)} ×100

##### The Morris-water Maze (MWM) test

The MWM test was conducted to evaluate memory and spatial learning in mice, using visual cues placed on the walls of a water tank. For five consecutive days, the mice were trained in the water maze, with each session involving the release of the mice from various starting points and the task of locating a hidden platform within the maze. Each day, the mice underwent four trials, following the established protocol by Charles V. Vorhees (2006). The time taken (latency) for each mouse to find the platform was measured and recorded throughout the training period.

On the sixth day, a probe test was conducted to assess reference memory. The hidden platform was removed, and the mice’s behavior was observed as they navigated the maze. The time spent in the target quadrant and the time taken to reach the former platform location were recorded. This probing phase aimed to evaluate the mice’s retention of the spatial information and memory acquired during the training sessions. The MWM test evaluated memory and spatial learning in mice using visual cues in a water tank. Over five days, mice were trained to find a platform within the tank, undergoing four trials daily as per Charles V. Vorhees' protocol. On day six, a probing experiment assessed reference memory. The platform was hidden with opaque water, and mice behavior was observed as they navigated the tank. Time spent and latency to reach the target quadrant were recorded. This phase gauged the retention of spatial information and memory acquired during training.

#### Parallel Artificial Membrane Permeation Assays assay

PAMPA assay was performed using a PAMPA filter plate (MAIPNTR10) coated with brain polar lipid extract (141101P) to evaluate blood-brain barrier (BBB) permeability of the compounds. The assay was performed with the help of the protocol provided by Merck Millipore on their official website and articles as cited.^62^^,^^63^ 5% of brain polar solution was used to coat the donor plate (15ul per well) by drying the plate in a fume hood. The acceptor plate was filled with 300ul of buffer (5% DMSO in PBS). The donor plate coated with brain polar solution was placed above the acceptor plate and different concentrations of Cdk5 inhibitor (100-500uM) dissolved in 5% DMSO were added to it (150ul per well). After 7 h of incubation, the absorbance was measured at broad spectrum 250–500 nm. The absorbance at 300nm was considered for further calculations as it showed an increasing pattern of ODs with an increase in the concentration of the inhibitors. For permeability, the P_app_ of the drugs was calculated.

P_aap_ = C ∗ -ln (1-[drug_acceptor_]/[Drug_equilibrium_]); C=V_D_∗V_A/_(V_D_ + V_A_) ∗ area ∗ time

P_aap_ < 10∗10^−6^ cm/s - Low permeability; P_aap_ > 10∗10^−6^ cm/s - high permeability

#### Western blotting

For tissue lysate preparation the mice were sacrificed following the Institution’s ethical guidelines. The hippocampus was isolated from the rest of the brain and treated in a tissue lysis solution with a protease and phosphatase inhibitor cocktail. Western blotting was performed to study the expression patterns of the protein of interest. Each well of a polyacrylamide gel was loaded with an identical amount of protein lysate (40–50 μg), which was then transferred to the nitrocellulose membrane. In 1X PBS with 0.1% (v/v) Tween 20, the main antibodies were diluted. (A list of the antibodies along with the dilutions utilized is provided in Table S4).

#### Immunohistochemistry

The brain stored in 4% formaldehyde was processed for parafilm block preparation. The brain embedded in paraffin blocks was cut into slices with a 5 μm thickness, which were then assembled on coated glass slides. The methods for single or double immunostaining were followed while doing immunohistochemistry.^14^ The primary antibodies utilized were NF-L(1:100), Tau(1:100), p-tau S396 (1:100), p-tau T-231 (1:100), and GFAP (1:300),. Utilizing secondary antibodies from Invitrogen’s Alexa fluor line that produce red (488 nm) and green (594 nm) light, immunostaining was seen.

#### The precise compound concentration determination in plasma and brain tissues using advanced mass spectrometry techniques

Metabolites, comprising the entire spectrum within the lysate excluding proteins but inclusive of drugs, were extracted from plasma and brain tissue homogenate samples. The extraction process involved precipitating proteins through the addition of methanol. In a concise procedure, 600 μL of methanol was introduced to 200 μL of the sample, followed by vortex mixing. The mixture was then incubated for 30 min in a −20°C freezer. This method facilitated the isolation of metabolites for subsequent analysis. The solution was centrifuged at 15000 g at 4°C for 10 min, and the supernatant was collected in a fresh tube and dried using a vacuum concentrator operated at room temperature. Extracted metabolites were reconstituted in 5 mL of 50% methanol with 0.1% formic acid and transferred to HPLC vials. Compounds were analyzed in these samples using a multiple reaction monitoring method (using parameters mentioned in Table S5) on a triple quadrupole hybrid ion trap mass spectrometer (QTRAP 6500+, SCIEX) coupled with an ExionLC UHPLC system (SCIEX). Optimized source and gas parameters were used and data acquisition was performed through Analyst 1.6.3 software in positive ion scan mode. 10 μL of metabolites suspension was loaded and resolved on an Acquity UPLC BEH C18 column (1.7 μm, 2.1 × 100 mm) using mobile phases of water with 0.1% formic acid (buffer A) and acetonitrile with 0.1% formic acid (buffer B) with a flow rate of 0.25 mL/min and 8 min long gradient. The gradient program was employed as follows: 2% buffer B for an initial 0.5 min that was ramped to 50% in the next 1.5 min. Buffer B was further increased to 90% in the next 1 min and kept constant at the same concentration for the next 2 min. Buffer B concentration was brought to an initial 2% in the next 1 min and the column was equilibrated at this buffer condition for 2 min before the next sample injection. Relative quantification was performed using MultiQuantTM software v.3.0 (SCIEX). A pooled QC from samples was used to analyze the technical variability and only metabolites with <30% coefficient of variation (%CV) were quantified.

#### Confocal microscope analysis

Confocal microscopy (Nikon Eclipse Ti2) was used to obtain the images of the IHC slides particularly sections from the hippocampus region. Z-stacks of successive confocal pictures are obtained by excitation using lasers (405-, 488-, and 561-lasers).

### Quantification and statistical analysis

Statistical analysis was conducted using GraphPad Prism 8. Data were presented as mean ± SD. One-way ANOVA with Tukey post hoc tests determined statistical significance between the means of all the independent groups. For the analyses of Cdk5 kinase assay (Figure 2H), GTT and ITT data between different groups at different time points (Figures 4A and 4B), and EPM data for both open and closed arms (Figure 4H) two-way ANOVA with Turkey post hoc test was used to compare between the groups. Statistical trends and test applied for analysis were indicated in figure legends, with *p*-values <0.05, <0.01, and <0.001 denoted by ∗, ∗∗, and ∗∗∗, respectively and ns denotes non-significant. A table (Table S6) lists all the exact *p*-values, number of biological units and degree of freedom for each group comparisons in all the graphs present in different figures with proper labeling. The information regarding the biological replicates is provided in each figure legends. The IC50 of inhibitors was calculated using the logarithm of x and normalized yvalues. Power calculations determined the sample size of *n* = 8 mice per group based on an estimated effect size (Cohen’s d = 1.2) from preliminary data, with 80% power and an alpha of 0.05. This size ensures detection of meaningful differences in behavioral outcomes while accounting for variability and adhering to ethical guidelines for minimizing animal use. Band intensities were measured using ImageJ software for Western blot analysis. The data were normalized to the signal from the protein loading control and expressed in arbitrary units.
