# Supplementary material for: A Cdk5 inhibitor restores cognitive function and alleviates type 2 diabetes in mice
Source: iScience. 2025 Mar 11;28(4):112200. doi: 10.1016/j.isci.2025.112200 (PMC11986975; doi:10.1016/j.isci.2025.112200)
Supplement: Document S1. Figures S1–S4 and Tables S1–S6 [file mmc1.pdf]

iScience, Volume *28*

Supplemental information

A Cdk5 inhibitor restores cognitive function

and alleviates type 2 diabetes in mice

Sangita Paul, Remya Chandran, Dileep K. Vijayan, Juhi Bhardwaj, Praveen Singh, Poornima Shetty, Srinivas Cheruku, Sajith Meleveetil, and Binukumar Balachandran Krishnamma

Supplementary tables and figures

Sl No	PDB ID	R-factor	R-Free	Ligand Name	Resolution (Å)	Space groups	Name used in the study
1	1UNH	0.23	0.23	L1	2.35	C 1 2 1	R1
2	1UNL	0.22	0.22	L2	2.20	P 32 2 1	R2
3	3O0G	0.23	0.26	L3	1.95	P 32 2 1	R3
4	4AU8	0.20	0.23	L4	1.90	P 41 21 2	R4

Table S1: Overview of the Cdk5 crystal structures employed in the screening of KINACore libraries.

Compounds	Smiles	MW
BLINK1	O=C([O-])CSc([nH]c1=O)nc(c12)sc3c2CC(C)(C)OC3	326.4
BLINK2	s1c(C)c(C)c(c12)c(=O)[nH]c(n2)SCC(=O)c3cc(C)c(cc3)OC	374.48
BLINK3	c1cccc(c12)oc(=O)c(c2)-c3nc(sc3)Cc4nc(cs4)-c5ccc(C)cc5	416.52

BLINK4	OCCNC(=O)c1c(ccc(Cl)c1)NC(=O)c2c(Cl)cc(Cl)cc2	387.65
BLINK5	C1CCCc(s2)c1c(c23)c(=O)[nH]c(n3)SCC(=O)N(C)C	323.44
BLINK6	c1cccc(Cl)c1-c2nc(NCCCO)nc(c23)ccc(Br)c3	392.69
BLINK7	c1cc(Cl)ccc1-c(sn2)nc2NC(=O)CSc3sc(nn3)C	383.9
BLINK8	c1c(C)ccc(c12)nc(nc2C)Nc3[nH]c(=O)cc(n3)CSc4sc(nn4)C	411.51
BLINK9	c1cccc(c12)oc(n2)Nc3nc(c(cn3)C(=O)OCC)CSc4sc(nn4)C	428.49
BLINK10	c1cccc(c12)oc(=O)c(c2)-c3nc(sc3)Cc4nc(cs4)-c5ccc(Cl)cc5	436.94
BLINK11	Clc1cccc(c1Cl)NC(=O)c2c(cccc2)OCC(=O)Nc(cc3)cc(Cl)c3N4CCOCC4	534.83
BLINK12	c1ccccc1-c2nc(nc(c23)ccc(Cl)c3)Nc(ccc4)cc4C(=O)NCCO	418.89
BLINK13	c1cccc(Cl)c1-c2nc([nH]n2)SCC(=O)c3cc(O)c(O)cc3	361.81
BLINK14	c1ccccc1Cc(s2)cc(C(=O)N)c2NC(=O)c3cc(nc(c34)c(	492.6

	C)cc(c4)C)-c5cnccc5	
BLINK15	COc1ccc(cc1[N+]([O-])=O)C(=O)C[C@@]2(O)C(=O)N(c(c23)ccc(Br)c3)Cc4ccccc4	511.33
BLINK16	c1c(Cl)ccc(c12)nc(o2)SCc(cs3)nc3Nc4ccccc4	373.89
BLINK17	c1cc(Cl)ccc1-c2nc(n[nH]2)SCC(=O)N[C@H]([C@H]34)C[C@@H](C3)CC4	362.88
BLINK18	c1ccc2cccc(c2c13)C(=O)N(C3=O)CCCSc(n[nH]4)nc4-c5ccc(F)cc5	432.48
BLINK19	ClCc1ncc2c(n1)sc3CC(C)CCc23	267.78

Table S2: The details of the 19 selected ligands obtained from screening of KINACore libraries against Cdk5

Compound	IC50 (nM) (Cdk5/p35)	IC50 (nM) Cdk5/p25

BLINK1	19.18	18.40
BLINK2	92.57	33.5
BLINK3	68.69	71.33
BLINK4	27.7	16.9
BLINK5	16.87	17.48
BLINK6	183	141
BLINK7	85.2	97
BLINK8	50.6	108.2
BLINK9	54.09	35.11
BLINK10	47.8	88.4

BLINK11	17.09	14.69
BLINK12	34.82	22.9
BLINK13	20.1	41.5
BLINK14	24.77	23.14
BLINK15	29.34	12.08
BLINK16	28.04	21.19
BLINK17	63.18	43.2
BLINK18	57.20	32.11
BLINK19	55.32	48.09

Table S3: The IC50 calculated based on the kinase assay of Cdk5/p35 and Cdk5p25 complexes with all the inhibitors.

Antibody	Dilution
Cdk5	1:1000
p35/25	1:1000
Neurofilament L	1:1000
Neurofilament H	1:1000
Tau	1:1000
p-Tau T-231	1:500
p-Tau S396	1:500
p-NF H	1:300
GFAP	1:1000; 1:200
Beta-actin	1:10,000
Beta tubulin	1:10,000

Table S4: **List of antibodies used in the study along with their dilutions used for experiments.**

Compound	Peak type	Precursor ion (m/z)	Fragment ion (m/z)	Collision energy (CE)	Declustering potential (DP)
BLINK11	Targeted ion	534.1	373.1	40	82
	Confirming ion	534.1	251.1	40	82
BLINK15	Targeted ion	511.1	105.2	23	76
	Confirming ion	511.1	316	23	76

Table S5: **Parameters used for compound screening in the MRM method**

Groups	Significance	Adjusted p-value	N	Degree of Freedom (DF)
**Figure 2H**				
w/o inhibitor vs. BLINK11-50	ns	0.9073	6	20
w/o inhibitor vs. BLINK11-100	**	0.0024	6	20
w/o inhibitor vs. BLINK15-50	ns	0.9998	6	20
w/o inhibitor vs. BLINK15-100	*	0.0259	6	20
**Figure 4C**				
Control vs. HFD	***	0.0001	8	48
HFD vs. HFD_BLINK11_20	**	0.0023	8	48
HFD vs. HFD_BLINK11_40	***	0.00021	8	48
HFD vs. HFD_BLINK15_20	**	0.0044	8	48
HFD vs. HFD_BLINK15_40	**	0.0016	8	48
**Figure 4E**				
Control vs. HFD	***	<0.0001	8	48
HFD vs. HFD_BLINK11_20	***	0.0003	8	48

HFD vs. HFD_BLINK11_40	***	0.0001	8	48
HFD vs. HFD_BLINK15_20	**	0.0032	8	48
HFD vs. HFD_BLINK15_40	***	0.0005	8	48
**Figure 4F**				
Control vs. HFD	**	0.0043	8	54
HFD vs. HFD_BLINK11_20	*	0.036	8	54
HFD vs. HFD_BLINK11_40	**	0.0075	8	54
HFD vs. HFD_BLINK15_20	ns	0.1645	8	54
HFD vs. HFD_BLINK15_40	ns	0.1978	8	54
**Figure 4G**				
Control vs. HFD	**	0.0049	8	42
HFD vs. HFD_BLINK11_20	**	0.0043	8	42
HFD vs. HFD_BLINK11_40	**	0.0056	8	42
HFD vs. HFD_BLINK15_20	ns	0.6132	8	42
HFD vs. HFD_BLINK15_40	**	0.0064	8	42
**Figure 4H**				
Control vs. HFD	**	0.0014	8	88
HFD vs. HFD_BLINK11_20	*	0.0364	8	88
HFD vs. HFD_BLINK11_40	*	0.0155	8	88
HFD vs. HFD_BLINK15_20	ns	0.9949	8	88
HFD vs. HFD_BLINK15_40	ns	0.5397	8	88
**Figure 5A**				
Control vs. HFD	**	0.0021	8	45
HFD vs. HFD_BLINK11_20	ns	0.1758	8	45
HFD vs. HFD_BLINK11_40	*	0.0346	8	45
HFD vs. HFD_BLINK15_20	ns	0.9761	8	45
HFD vs. HFD_BLINK15_40	ns	0.8525	8	45
**Figure 5C**				
Control vs. HFD	ns	0.711	8	41
HFD vs. HFD_BLINK11_20	ns	0.98	8	41
HFD vs. HFD_BLINK11_40	ns	>0.9999	8	41
HFD vs. HFD_BLINK15_20	ns	0.9986	8	41
HFD vs. HFD_BLINK15_40	ns	>0.9999	8	41
**Figure 5E**				
Control vs. HFD	**	0.002	8	52
HFD vs. HFD_BLINK11_20	**	0.0032	8	52
HFD vs. HFD_BLINK11_40	***	0.0003	8	52
HFD vs. HFD_BLINK15_20	*	0.0253	8	52
HFD vs. HFD_BLINK15_40	**			
**Figure 6B**				
Control vs. HFD	ns	0.7591	6	30

HFD vs. HFD_BLINK11_20	ns	0.9994	6	30
HFD vs. HFD_BLINK11_40	ns	0.9115	6	30
HFD vs. HFD_BLINK15_20	ns	0.99	6	30
HFD vs. HFD_BLINK15_40	ns	0.9964	6	30
**Figure 6C**				
Control vs. HFD	**	0.0057	6	30
HFD vs. HFD_BLINK11_20	ns	0.7818	6	30
HFD vs. HFD_BLINK11_40	ns	0.2169	6	30
HFD vs. HFD_BLINK15_20	ns	0.9882	6	30
HFD vs. HFD_BLINK15_40	ns	0.443	6	30
**Figure 6D**				
Control vs. HFD	***	0.0001	6	30
HFD vs. HFD_BLINK11_20	*	0.0265	6	30
HFD vs. HFD_BLINK11_40	**	0.005	6	30
HFD vs. HFD_BLINK15_20	ns	0.4837	6	30
HFD vs. HFD_BLINK15_40	*	0.0212	6	30
**Figure 7B**				
Control vs. HFD	*	0.0346	6	24
HFD vs. HFD_BLINK11_20	ns	0.8441	6	24
HFD vs. HFD_BLINK11_40	*	0.0238	6	24
HFD vs. HFD_BLINK15_20	ns	0.9994	6	24
HFD vs. HFD_BLINK15_40	ns	0.835	6	24
**Figure 7C**				
Control vs. HFD	*	0.0205	6	30
HFD vs. HFD_BLINK11_20	ns	0.7329	6	30
HFD vs. HFD_BLINK11_40	ns	0.301	6	30
HFD vs. HFD_BLINK15_20	ns	0.8616	6	30
HFD vs. HFD_BLINK15_40	ns	0.9748	6	30
**Figure 7D**				
Control vs. HFD	**	0.0044	6	30
HFD vs. HFD_BLINK11_20	ns	0.7129	6	30
HFD vs. HFD_BLINK11_40	*	0.0136	6	30
HFD vs. HFD_BLINK15_20	ns	0.975	6	30
HFD vs. HFD_BLINK15_40	ns	>0.9999	6	30
**Figure 7E**				
Control vs. HFD	***	0.0001	6	30
HFD vs. HFD_BLINK11_20	ns	0.3789	6	30
HFD vs. HFD_BLINK11_40	***	0.0002	6	30
HFD vs. HFD_BLINK15_20	ns	0.6538	6	30
HFD vs. HFD_BLINK15_40	ns	0.084	6	30
**Figure 7F**				

Control vs. HFD	ns	>0.9999	6	30
HFD vs. HFD_BLINK11_20	ns	0.9526	6	30
HFD vs. HFD_BLINK11_40	ns	>0.9999	6	30
HFD vs. HFD_BLINK15_20	ns	>0.9999	6	30
HFD vs. HFD_BLINK15_40	ns	0.9923	6	30
**Figure 7G**				
Control vs. HFD	**	0.003	6	30
HFD vs. HFD_BLINK11_20	ns	0.9982	6	30
HFD vs. HFD_BLINK11_40	*	0.0107	6	30
HFD vs. HFD_BLINK15_20	ns	0.6255	6	30
HFD vs. HFD_BLINK15_40	ns	0.9773	6	30
**Figure 7H**				
Control vs. HFD	**	0.0057	6	30
HFD vs. HFD_BLINK11_20	ns	0.4325	6	30
HFD vs. HFD_BLINK11_40	**	0.0041	6	30
HFD vs. HFD_BLINK15_20	ns	0.6981	6	30
HFD vs. HFD_BLINK15_40	ns	0.4045	6	30
**Figure 8D: p-tau 231**				
Control vs. HFD	ns	0.109	6	20
HFD vs. HFD_BLINK11_20	ns	0.8024	6	20
HFD vs. HFD_BLINK11_40	ns	0.8189	6	20
**Figure 8D: p-tau 396**				
Control vs. HFD	**	0.0074	6	20
HFD vs. HFD_BLINK11_20	*	0.9537	6	20
HFD vs. HFD_BLINK11_40	*	0.0107	6	20
**Figure 8D: Tau**				
Control vs. HFD	*	0.0333	6	20
HFD vs. HFD_BLINK11_20	ns	0.687	6	20
HFD vs. HFD_BLINK11_40	ns	0.8722	6	20
**Figure 8E**				
Control vs. HFD	*	0.0124	6	20
HFD vs. HFD_BLINK11_20	ns	0.9997	6	20
HFD vs. HFD_BLINK11_40	*	0.0281	6	20
**Figure 8F**				
Control vs. HFD	***	0.0003	6	20
HFD vs. HFD_BLINK11_20	ns	0.1069	6	20
HFD vs. HFD_BLINK11_40	**	0.005	6	20

Table S6: List of statistical analysis of the graphs: A table presenting the precise p-values, the number of biological units (N), and the degrees of freedom (DF) used in the statistical analysis for group comparisons.

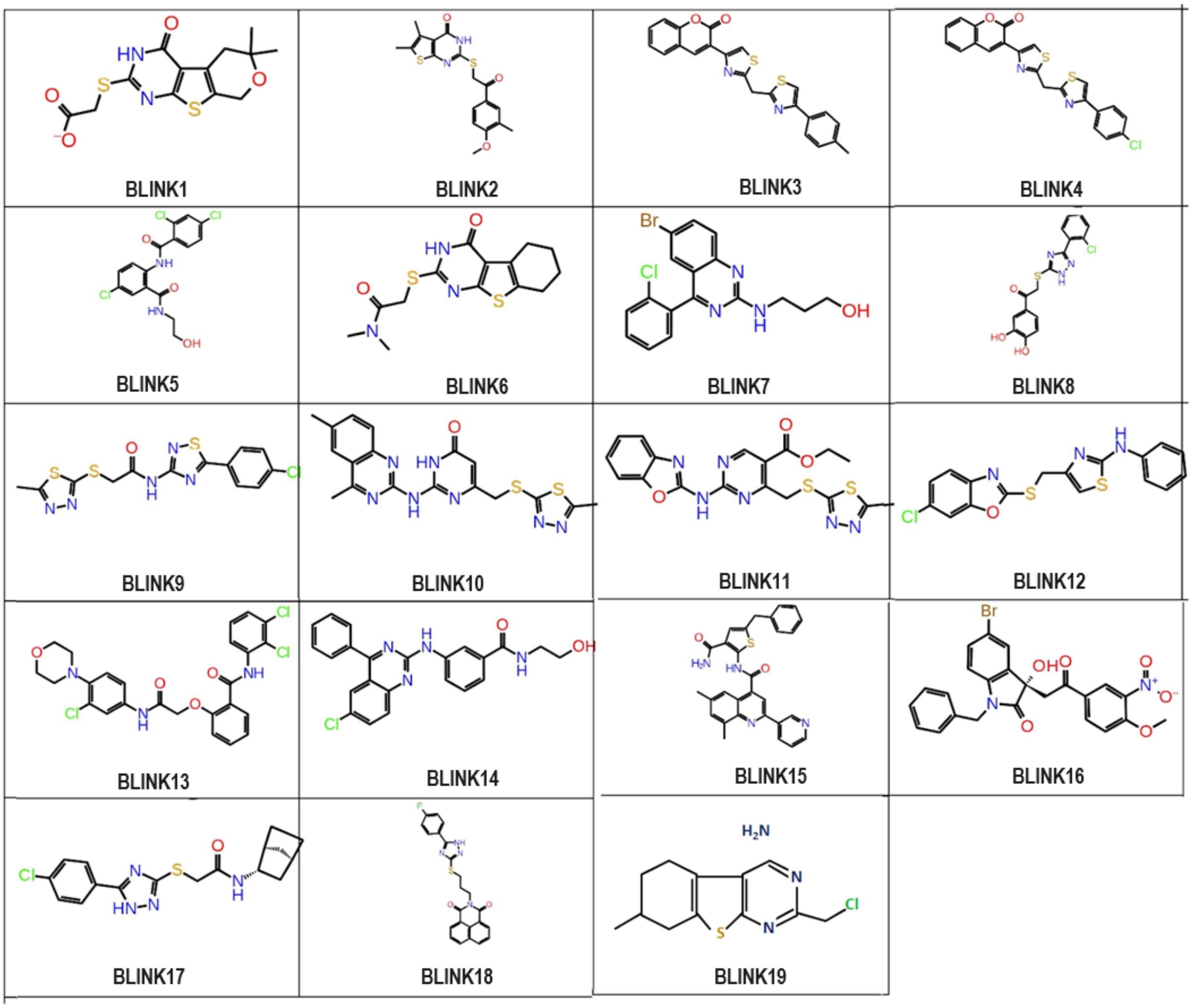

Figure S1: Structure of all the 19 compounds (BLINK1-19) selected by *in silico* approach

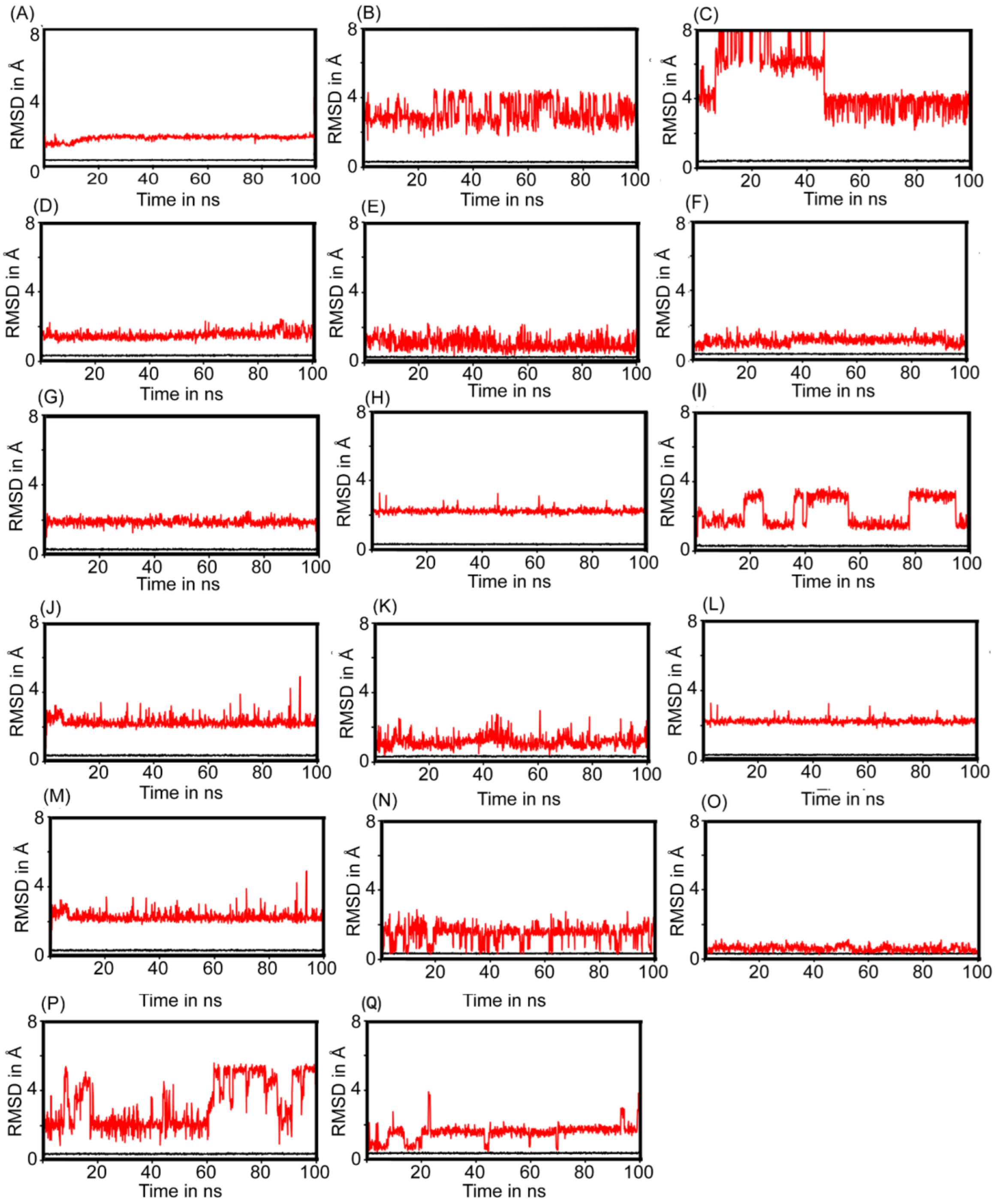

Figure S2: RMSD plot determined for Cdk5-BLINK complexes (A-Q) BLINK1-19 respectively. In each complex, Cdk5 is denoted by black lines, while the corresponding

ligands are illustrated with red lines. The RMSD plots for the interactions of BLINK11 and BLINK15 with Cdk5 are individually displayed in Figures 2F and 2G, respectively.

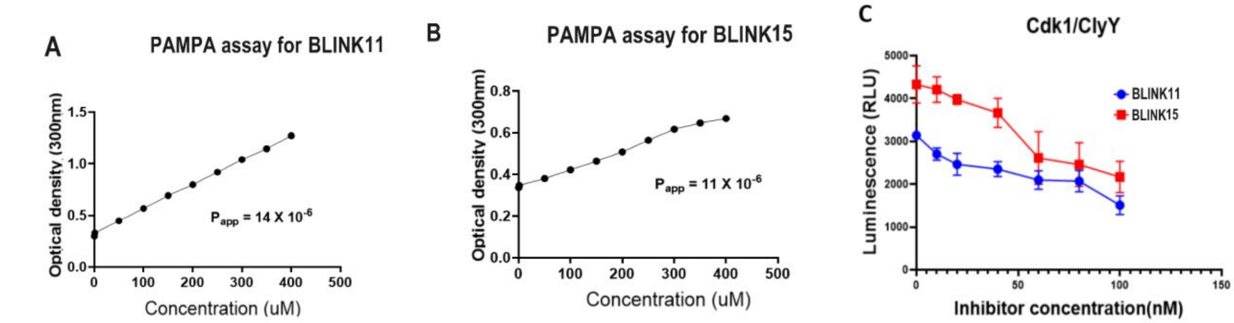

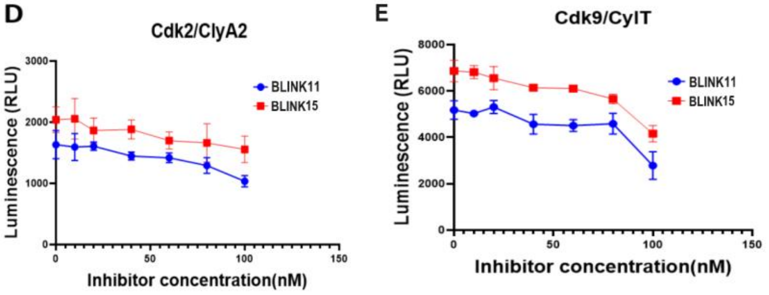

F

Kinases	IC50 for BLINK11 (nM)	IC50 for BLINK15 (nM)
Cdk1/ClyY	39.34	35.84
Cdk2/ClyA2	63.43	44.12
Cdk9/CylT	83.43	74.63

Figure S3: BBB Permeability assay and Evaluation of non-specific kinase inhibition by BLINK11 and BLINK15 (A-B) PAMPA assay results of BLINK11 and BLINK15 compound. The graph shows the inhibition pattern of (C) Cdk1/CylY kinase activity (D) Cdk2/CylA2 and (E) Cdk9/CylT by BLINK11 and BLINK15 with increasing concentration (0-100nM) (F) Shows IC50 of all the kinases for both BLINK11 and BLINK15 inhibitors.

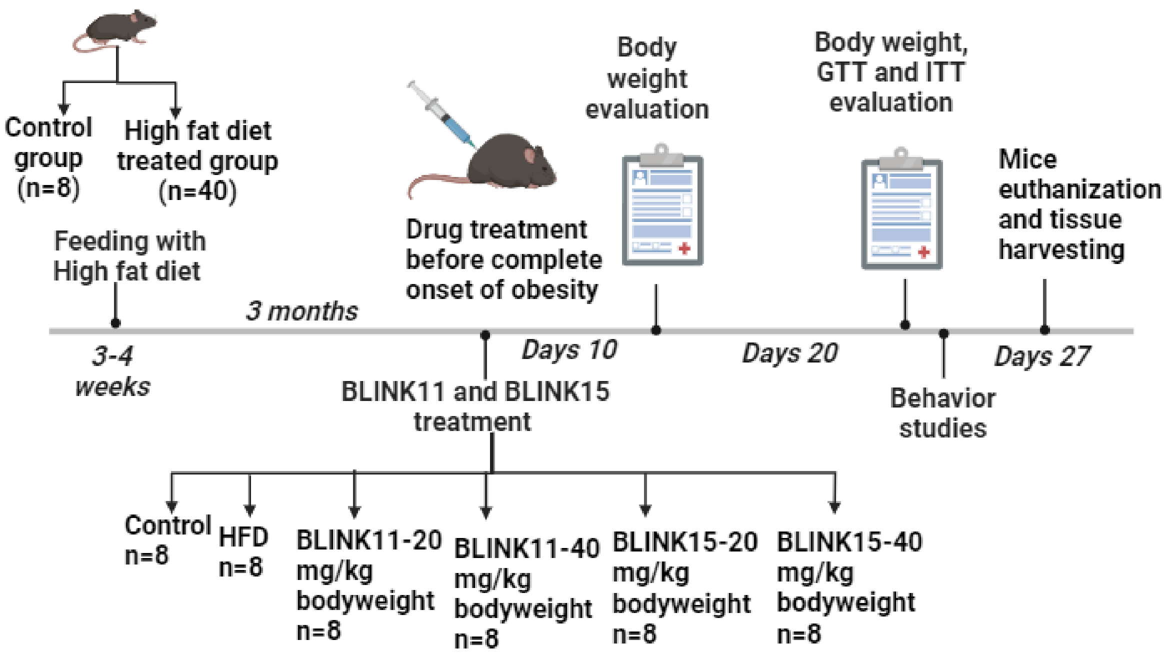

Figure S4: The flowchart of HFD mice model generation and drug treatment.
